# Biomarker LEPRE1 induces pelitinib-specific drug responsiveness by regulating ABCG2 expression and tumor transition states in human leukemia and lung cancer

**DOI:** 10.1038/s41598-022-06621-w

**Published:** 2022-02-21

**Authors:** A-Ram Lee, Sunho Lee, Jee Yoon Shin, Ji-Young Kim, Kyoung-Sik Moon, Joungsun Jung

**Affiliations:** 1 Department of Advanced Toxicology, Korea Institute of Toxicology (KIT), Daejeon, 34114 Republic of Korea; 2 Present Address: Bio Center, LegoChem Biosciences, Inc., Daejeon, 34002 Republic of Korea; 3 Genome Data Integration Centre, Syntekabio Inc., Daejeon, 34025 Republic of Korea; 4 Present Address: Department of Computer Science and Engineering, Seoul National University, Seoul, 08826 Republic of Korea

**Keywords:** Cancer, Cell biology, Computational biology and bioinformatics, Biomarkers, Oncology

## Abstract

Biomarkers for treatment sensitivity or drug resistance used in precision medicine include prognostic and predictive molecules, critical factors in selecting appropriate treatment protocols and improving survival rates. However, identification of accurate biomarkers remain challenging due to the high risk of false-positive findings and lack of functional validation results for each biomarker. Here, we discovered a mechanical correlation between leucine proline-enriched proteoglycan 1 (LEPRE1) and pelitinib drug sensitivity using *in silico* statistical methods and confirmed the correlation in acute myeloid leukemia (AML) and A549 lung cancer cells. We determined that high LEPRE1 levels induce protein kinase B activation, overexpression of ATP-binding cassette superfamily G member 2 (ABCG2) and E-cadherin, and cell colonization, resulting in a cancer stem cell-like phenotype. Sensitivity to pelitinib increases in LEPRE1-overexpressing cells due to the reversing effect of ABCG2 upregulation. LEPRE1 silencing induces pelitinib resistance and promotes epithelial-to-mesenchymal transition through actin rearrangement via a series of Src/ERK/cofilin cascades. The *in silico* results identified a mechanistic relationship between LEPRE1 and pelitinib drug sensitivity, confirmed in two cancer types. This study demonstrates the potential of LEPRE1 as a biomarker in cancer through in-silico prediction and in vitro experiments supporting the clinical development of personalized medicine strategies based on bioinformatics findings.

## Introduction

According to the United States National Institutes of Health (NIH), precision medicine is “an emerging approach for disease treatment and prevention accounting for individual variability in genes, environment, and lifestyle”^1^. Following the announcement of the Precision Medicine Initiative, there has been a paradigm shift away from the traditional “one-size-fits-all” approach, along with raised hopes of providing new therapies for generations of patients to come^2^.

Recently, biomarker discovery has been enhanced by high-throughput pharmacogenomic studies using large panels of cancer-derived cell lines. The GDSC database has successfully identified drug sensitivity biomarkers that are observed clinically^3^. As a result, more than 25 molecular therapies in 526 forms of cancer have been approved for clinical use based on predictive biomarkers^4^. However, significant limitations remain, including the low statistical power and limited ability to integrate results due to differences in the types of assays, the maximum and range of drug concentrations tested, and the drug sensitivity metrics employed by different studies^5,6^.

Leucine-proline-enriched proteoglycan 1 (LEPRE1), also known as Gros1 and P3H1, belongs to the prolyl 3-hydroxylase family and has the necessary function of proline hydroxylation in collagen synthesis^7^. Hydroxylation of type I collagen by LEPRE1 is related to bone metastasis, and LEPRE1 expression levels are increased in solid tumors, such as pancreatic, colorectal, breast, and lung cancers^8–10^. LEPRE1 has also been shown in osteosarcoma cell lines involved in cell proliferation, migration, and invasion^11^.

Epidermal growth factor receptor (EGFR) is a member of the ErbB family of receptor tyrosine kinases and consists of four members, ErbB-1 (EGFR), ErbB-2 (HER2/neu), ErbB-3 (HER3), and ErbB-4 (HER4)^12^. EGFR activation leads to downstream stimulation of multiple signaling cascades, including MAPK and phosphoinositide 3-kinases (PI3K)/protein kinase B (AKT), which are tightly associated with oncogenesis, proliferation, maintenance, migration, and survival of cancer cells^13–15^. Consequently, EGFR signaling overactivation has been detected in various malignant tumors, including non-small cell lung cancer (NSCLC), colon, head and neck, breast, and ovarian cancer^16–18^. For this reason, EGFR activation and subsequent intracellular signaling molecules have long been attractive candidates as anticancer drug targets^19^.

There are currently three generations of EGFR tyrosine kinase inhibitors (TKIs), namely, first-generation agents erlotinib and gefitinib, the second-generation ErbB family blocker afatinib, and pelitinib (EKB-569), and third-generation osimertinib^20^. The second-generation EGFR-TKI pelitinib is a potent, low-molecular-weight, selective, irreversibly binding inhibitor of EGFR TK activity^21^. In contrast to other EGFR-TKIs, pelitinib targets upstream molecules, such as EGFR signaling and HER-2, and downstream components such as Src, MEK/ERK, and Raf^22,23^.

In the current study, we investigated pelitinib sensitivity and determined the expression of LEPRE1 in AML cell lines and lung cancer cell line A549, referring to biomechanical information derived from in-silico analyses. We also genetically manipulated the level of LEPRE1 expression in leukemia and lung cancer cells and revealed that LEPRE1 overexpression led to increased pelitinib sensitivity via AKT activation and overexpression of ATP-binding cassette superfamily G member 2 (ABCG2). In contrast, LEPRE1 knockdown induced EGFR/Src/ERK/cofilin cascade signaling and promoted epithelial-to-mesenchymal transition (EMT), leading to pelitinib resistance in the AML cell line THP-1 and lung cancer-derived A549 cells.

## Results

### Biomarkers in blood cancer cells screened for six EGFR signaling pathway drugs

**Figure 1:**
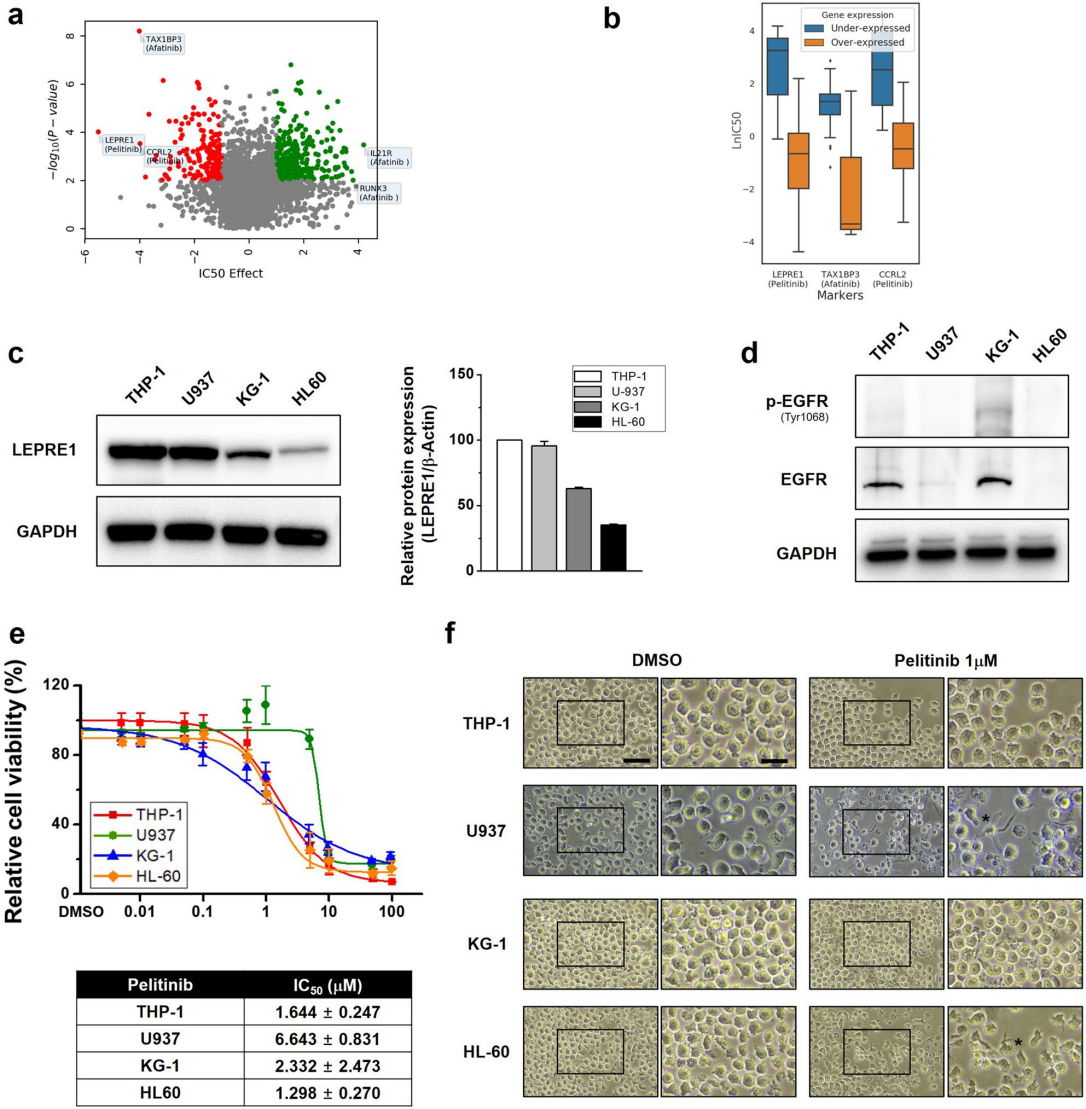
LEPRE1 in blood cancer cells is a potential biomarker for pelitinib treatment. **(a**) Volcano plot showing the variation in IC_50_ effect for the drugs and genes indicated. The significance (p-value) between drug responsiveness and the gene expression level was calculated using a t-test. (**b**) Box plots show the IC_50_ values of the drugs according to gene expression levels of the samples. (**c**) Differential expression levels of LEPRE1 and pelitinib sensitivity in AML cell lines. LEPRE1 protein levels in AML cells were evaluated by immunoblotting using a LEPRE1-specific antibody. (**d**) Absence of EGFR expression in AML-derived U937 and HL60 cells determined by immunoblotting. (**e**) WST-1 proliferation assay with pelitinib. AML cells were treated with the indicated concentration of pelitinib for 72 h, and the WST-1 assay was used to assess cell viability. Data are shown as the mean ± SEM (n = 3). Red line: THP-1 cells, green line: U937 cells, blue line: KG-1 cells, orange line: HL60 cells. The IC_50_ of each cell line is shown in the lower panel. (**f**) Effect of DMSO or pelitinib (1 μM) on cell morphology. The AML cell lines were treated with DMSO or pelitinib for 72 h. Undifferentiated cells and differentiated cells (asterisks) are shown in a selected enhanced micrograph on the right panel of each picture. The asterisks (*) indicates differentiated cell morphology. Scale bar, 100 μm (low magnification), 50 μm (high magnification).

To select candidate genes in blood cancer cells associated with EGFR signaling pathway drugs, data on 167 blood cancer cell lines from GDSC1 and six EGFR signaling pathway drugs were analyzed. We found that LEPRE1 or CCRL2 overexpression was associated with pelitinib sensitivity, and TAX1BP3 overexpression was associated with afatinib sensitivity (Fig. 1a). A positive IC_50_ value indicated that a particular gene was associated with increased drug resistance, whereas a negative value indicated increased drug sensitivity.

Although statistical analyses of the entire samples are generally reliable, it is also important to consider the drug responsiveness of differentially expressed samples. We used differentially expressed samples to prioritize the candidates. Figure 1b shows the difference in drug responsiveness between the two groups of cells where a gene was overexpressed in one group (z-score > 1) and under-expressed in the other (z-score < − 1). Under-expression of IL21R or RUNX3 was associated with afatinib resistance. The association between LEPRE1 and pelitinib, which exhibited the highest IC_50_, was further evaluated using drug responsiveness and mechanism studies.

### LEPRE1 expression and pelitinib sensitivity differ in AML cell lines

To examine LEPRE1 expression in AML cells, we evaluated expression levels in four different AML-derived cell lines, THP-1, U937, KG-1, and HL60 (Fig. 1c). LEPRE1 was strongly expressed in THP-1 and U937 cells, however, a weaker expression was detected in KG-1 and HL60 cells. We then confirmed protein expression levels of EGFR in the AML cell lines. EGFR protein was observed in only THP-1 and KG-1 cells (Fig. 1d).

Recently, there have been several reports on the ability of EGFR inhibitors and EGFR-TKIs to induce differentiation or death of AML cells, which is generally considered an off-target effect^25,26^. To determine whether pelitinib affected AML cell viability, we exposed the cells to pelitinib at concentrations ranging from 0 to 10 μM for 72 h. A dose-dependent decrease in cell viability was observed for the EGFR-positive cell lines THP-1 and KG-1, but not for U937 or HL60 cell lines (Fig. 1e). When U937 and HL60 cells were treated with pelitinib, differentiated cell morphology was observed (Fig. 1f). From these results, we selected the KG-1 and THP-1 cell lines for gain-of-function and loss-of-function evaluation, respectively.

### Verification of bioinformatics prediction regarding LEPRE1 and pelitinib drug responsiveness in AML

To elucidate the effects of LEPRE1 expression in AML, siRNAs or plasmid DNA were electroporated into THP-1 and KG-1 cells. First, we investigated the efficacy of siRNAs against endogenous LEPRE1 in THP-1 cells. Based on quantitative immunoblotting results, si2293 suppressed LEPRE1 expression to 83% of the negative control (Supplementary Fig. 1a). The ectopic expression of LEPRE1/pcDNA3.1 significantly increased the LEPRE1 protein levels in the KG-1 cell line compared to the vector control (Supplementary Fig. 1b).

**Figure 2:**
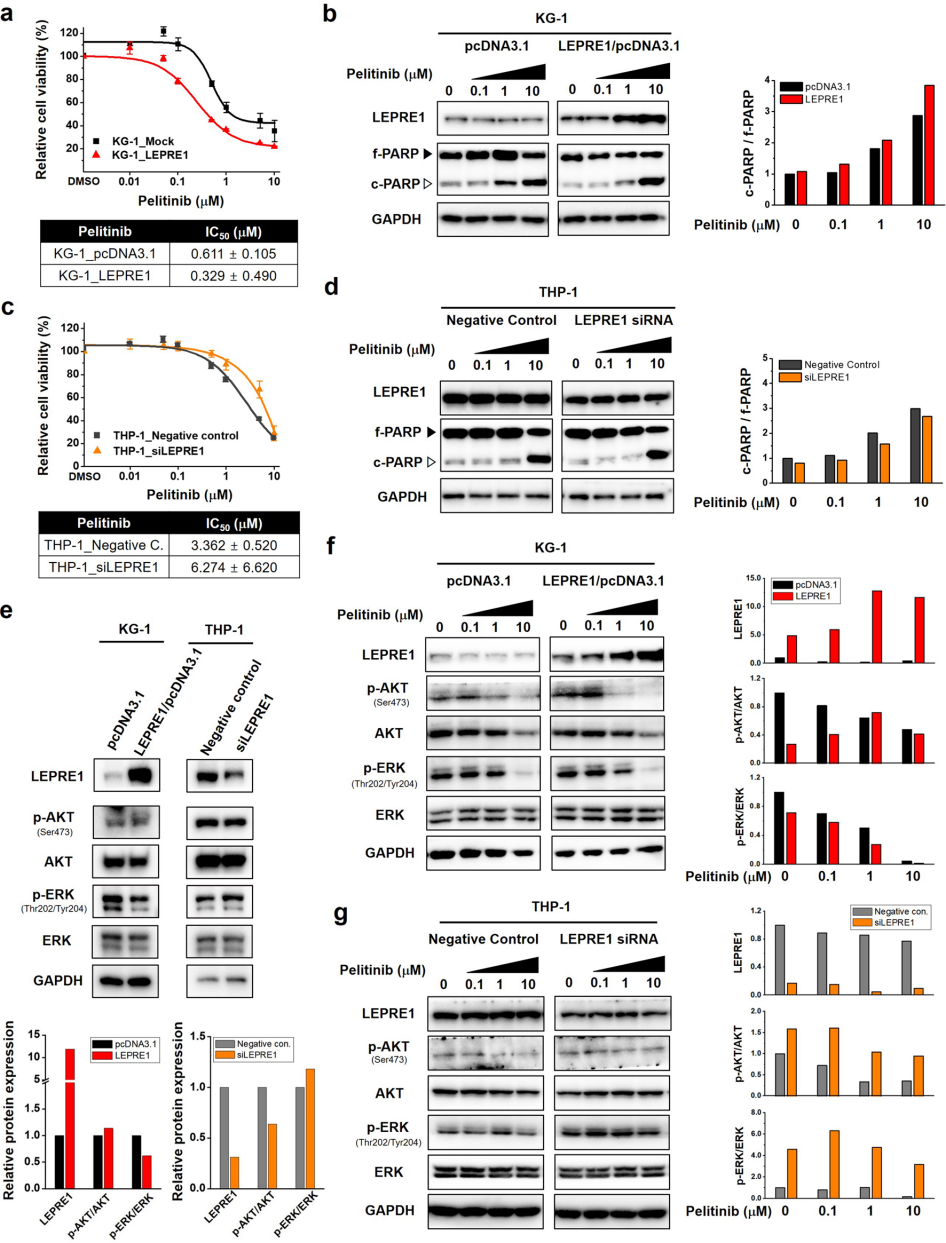
LEPRE1 expression levels in AML-derived cell lines regulate cell survival against pelitinib. **(a,c**) Transfected KG-1 cells (**a**) and THP-1 cells (**c**) treated with pelitinib for 3 days. Cell viability was determined using a WST-1 proliferation assay. (**b**,**d**) Overexpression of LEPRE1 in KG-1 cells (**b**) and knockdown of LEPRE1 in THP-1 cells (**d**) treated with 0, 0.1, 1, 10 μM of pelitinib for 24 h. Whole-lysates extracted from these cells were subjected to western blot analysis with PARP antibody. Band intensities were quantified by densitometric analysis and normalized by GAPDH. f-PARP, full-length PARP; c-PARP, cleaved PARP. (**e**) KG-1 and THP-1 cells transfected with pcDNA3.1 and LEPRE1/pcDNA3.1 or with Negative control siRNA and LEPRE1 siRNA, as indicated. After 48 h, p-AKT, AKT, p-ERK, and ERK expression levels were determined by western blot analysis. (**f**,**g**) KG-1 and THP-1 cells were transfected with pcDNA3.1 and LEPRE1/pcDNA3.1 (**f**) or Negative control siRNA and LEPRE1 siRNA (**g**) and then treated with the indicated concentration of pelitinib (0, 0.1, 1, 10 μM) for 24 h. Cell extracts were prepared and immunoblotted using the indicated antibodies. GAPDH was used as an internal control. All western blots are pre-cut. Membranes were often stripped and reprobed for multiple antibodies.

To examine the role of LEPRE1 in pelitinib cytotoxicity, cells transfected with LEPRE1 (KG-1_pcDNA3.1, KG-1_LEPRE1, THP-1_NC, and THP-1_siLEPRE1) were treated with pelitinib. Analyses of pelitinib cytotoxicity and expression of cleavage-PARP (c-PARP) revealed that LEPRE1 overexpression resulted in increased pelitinib sensitivity compared with that of control cells receiving the empty vector (Fig. 2a,b). In contrast, THP-1_siLEPRE1 transformed cells showed resistance to pelitinib compared to the negative control cells (Fig. 2c,d).

We evaluated the influence of LEPRE1 on the EGFR signaling pathway in KG-1 and THP-1 cells for functional analyses and found that p-ERK in the LEPRE1-overexpression group shared a repressive tendency compared with that of KG-1 control cells transformed with empty vector. In the siLEPRE1 group, p-ERK levels were increased compared to the THP-1 negative control cells (Fig. 2e). These data demonstrated that LEPRE1 might be related to the ERK signaling pathway.

Next, we assessed the effect of pelitinib on the EGFR signaling pathways in these cell lines. Whole-cell lysates from KG-1 and THP-1 cells were evaluated for total EGFR, AKT, and ERK levels by western blotting after 24 h of exposure to pelitinib. In KG-1_LEPRE1 cells, p-ERK levels were reduced in a pelitinib dose-dependent manner (Fig. 2f). The resistance of p-ERK was observed in THP-1_LEPRE1 cells (Fig. 2g). These results indicate that LEPRE1 inhibition promoted pelitinib resistance, whereas LEPRE1-overexpression induced high pelitinib sensitivity.

### LEPRE1 expression is associated with pelitinib-specific sensitivity/resistance in AML

**Figure 3:**
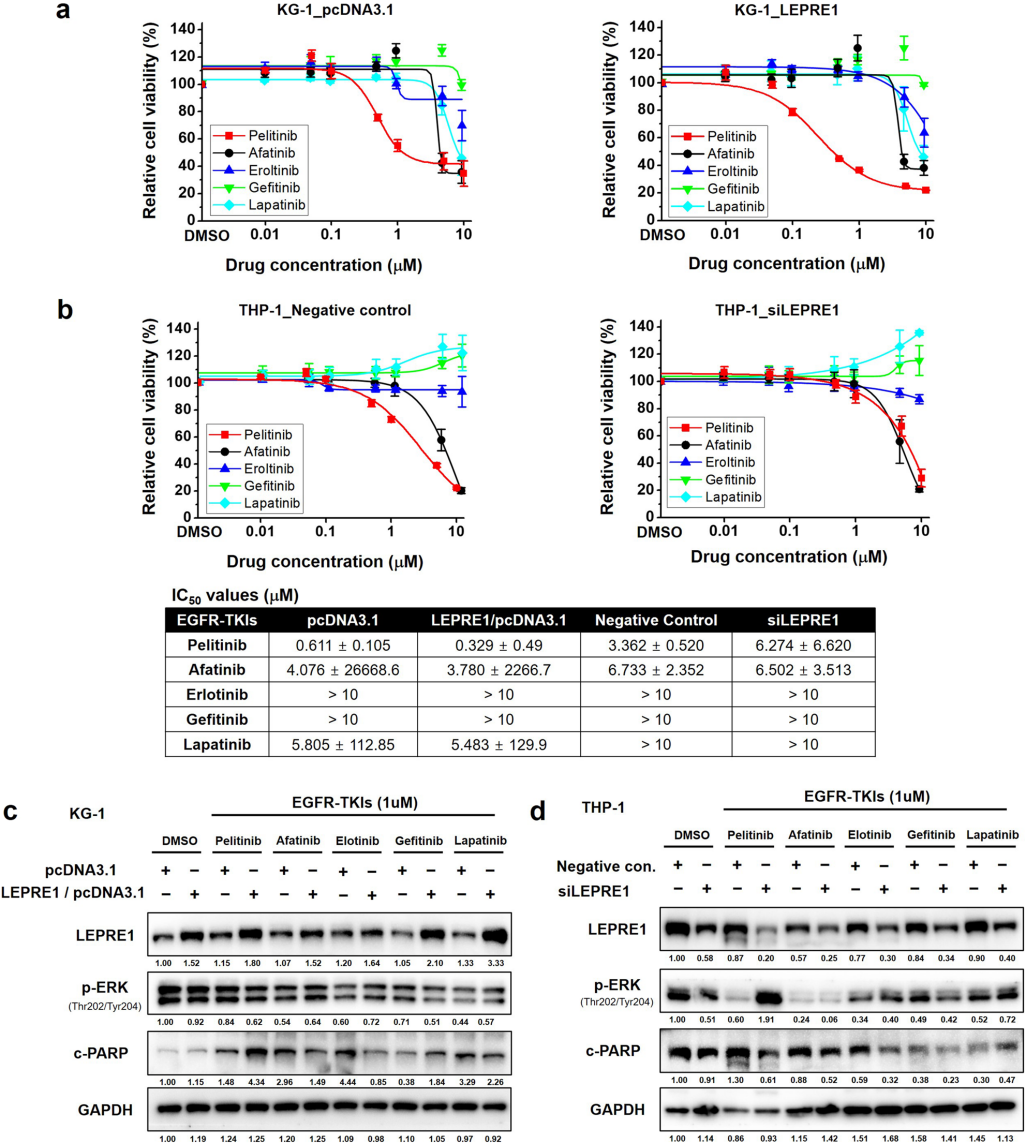
Effect of EGFR-TKIs on LEPRE1 overexpression or knockdown AML cells. **(a**,**b**) pcDNA3.1 and LEPRE1/pcDNA3.1 were transfected into KG-1 cells (**a**) or Negative control siRNA, and siLEPRE1 were transfected into THP-1 cells (**b**) and then incubated with EGFR-TKIs (afatinib, erlotinib, gefitinib, lapatinib, and pelitinib) for 72 h. Cell viability was determined using WST-1 proliferation assays. The IC_50_ value for each condition is presented in the lower panel. (**c**,**d**) Transfected cells were incubated with EGFR-TKIs (1 μM) for 24 h. Thereafter, cell lysates were prepared and immunoblotted for phosphorylated ERK (p-ERK) and cleaved PARP (c-PARP), as indicated. All western blots are pre-cut. Membranes were often stripped and reprobed for multiple antibodies.

We then examined the chemosensitizing effect of various EGFR-TKIs (afatinib, erlotinib, gefitinib, lapatinib, and pelitinib) in AML cell lines and investigated the association between LEPRE1 expression levels and sensitivity to EGFR-TKI treatment (Supplementary Fig. 2). Pelitinib produced the most dramatic inhibitory effect on KG-1_LEPRE1 cells, which had an IC_50_ value of 0.329 μM compared to KG-1_pcDNA3.1 cells. Afatinib and lapatinib both inhibited the proliferation of KG-1_LEPRE1 cells, although they were less potent than pelitinib (Fig. 3a). Erlotinib and gefitinib exhibited IC_50_ values of more than 10 μM and failed to inhibit proliferation of either pcDNA3.1-transfected or LEPRE1-transfected KG-1 cells. Only THP-1_ siLEPRE1 showed resistance to pelitinib compared to negative controls (Fig. 3b). Erlotinib, gefitinib, and lapatinib had IC_50_ values exceeding 10 μM in both negative control and LEPRE1 knockdown THP-1 cells and failed to inhibit proliferation.

Immunoblotting was performed to determine whether sensitivity to EGFR-TKIs was mediated through EGFR signaling inhibition. Consistent with the proliferation results of the WST-1 assays, pelitinib-treated KG-1_LEPRE1 cells demonstrated decreased p-ERK and increased c-PARP levels (Fig. 3c). Meanwhile, THP-1_ siLEPRE1 cells showed increased p-ERK expression and decreased c-PARP compared with the THP-1_Negative control cells (Fig. 3d). These results suggest that LEPRE1 expression was associated with pelitinib-specific sensitivity or resistance in AML cells, consistent with the bioinformatics data shown in Fig. 1a,b.

### LEPRE1 contributes to pelitinib responsiveness in A549 cells via EGFR-independent signaling

Pelitinib is a potent irreversible EGFR-TKI that has been evaluated in clinical trials for the treatment of lung cancer^27,28^. As there are many studies regarding EGFR-TKI targeting NSCLC, we verified the drug responsiveness of pelitinib relative to LEPRE1 levels in EGFR-expressing human lung cancer-derived A549 cells.

**Figure 4:**
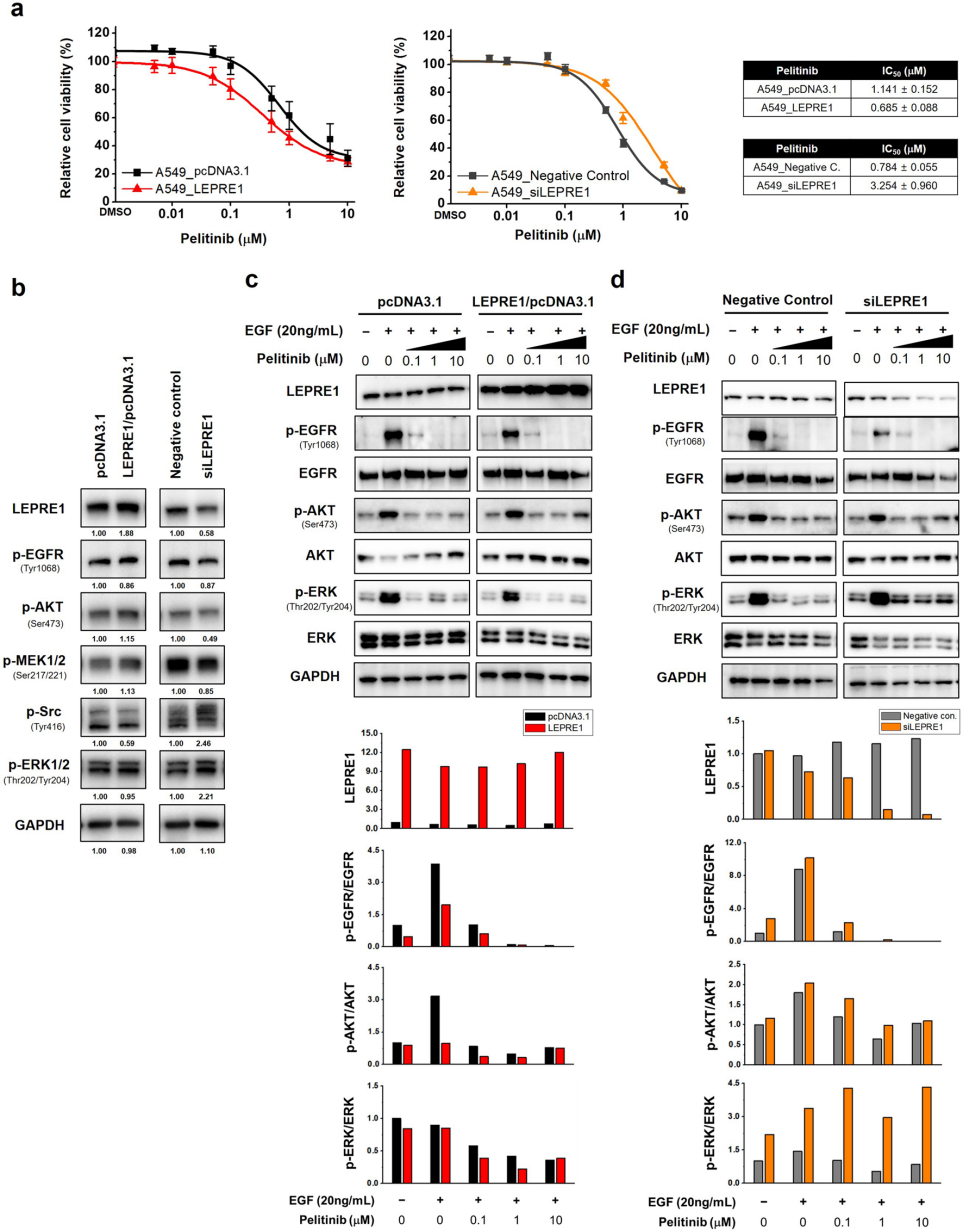
LEPRE1 regulates drug responsiveness in lung cancer-derived A549 cells. **(a**) LEPRE1 overexpression (left side) or LEPRE1 knockdown (right side) A549 cells were treated with pelitinib for 3 days. Cell viability was determined using WST-1 proliferation assays. (**b**) A549 cells were transfected with pcDNA3.1 and LEPRE1/pcDNA3.1 or with Negative control siRNA and LEPRE1 siRNA, as indicated. After 48 h, EGFR, AKT, and ERK expression were determined by western blot analysis. (**c**,**d**) A549 cells were transfected with pcDNA3.1 and LEPRE1/pcDNA3.1 (**c**) or with Negative control siRNA and LEPRE1 siRNA (**d**) and then treated with EGF and/or the indicated concentration of pelitinib (0, 0.1, 1, 10 μM) for 24 h. Cell extracts were prepared and immunoblotted using the indicated antibodies. GAPDH was used as an internal control. All western blots are pre-cut. Membranes were often stripped and reprobed for multiple antibodies.

To evaluate pelitinib cytotoxicity, LEPRE1 overexpression or knockdown A549 cells were treated with pelitinib. Pelitinib-induced sensitivity in the A549 cells was consistent with the AML-based results (Fig. 2a,c). LEPRE1 overexpression correlated with greater pelitinib sensitivity (Fig. 4a). In contrast, LEPRE1-knockdown A549 cells showed greater resistance to pelitinib compared to the negative control cells.

Expression of downstream signaling molecules in A549 cells changed according to the level of LEPRE1 expression (Fig. 4b). Similar to the AML results, LEPRE1 regulated AKT/ERK and EGFR activity in A549 cells. To confirm the EGFR pathway inhibition by pelitinib treatment, transfected A549 cells were treated with hEGF after 24 h pre-treatment with pelitinib. EGF treatment stimulated EGFR phosphorylation and increased AKT and ERK phosphorylation. However, when cells were pre-treated with pelitinib, EGFR phosphorylation was inhibited in both A549_LEPRE1 and A549_siLEPRE1 cells in a dose-dependent manner (Fig. 4c,d). In addition, reduced p-ERK was detected in LEPRE1-overexpressing A549 cells in pelitinib dose-dependently (Fig. 4c). However, a decrease in p-ERK relative to pelitinib concentration was not observed in LEPRE1-knockdown cells (Fig. 4d). These results support that LEPRE1 regulated ERK activation via an EGFR-independent pathway. In conclusion, the loss of LEPRE1 expression decreased pelitinib-induced apoptosis and promoted pelitinib resistance in A549 cells via the activation of ERK. Furthermore, the therapeutic efficacy of pelitinib increased in A549 cells in conjunction with higher LEPRE1 expression. Pelitinib-specific drug sensitivity was also observed in A549 cells (Supplementary Fig. 3).

### LEPRE1 affects cell motility and cell morphology of A549 cells

**Figure 5:**
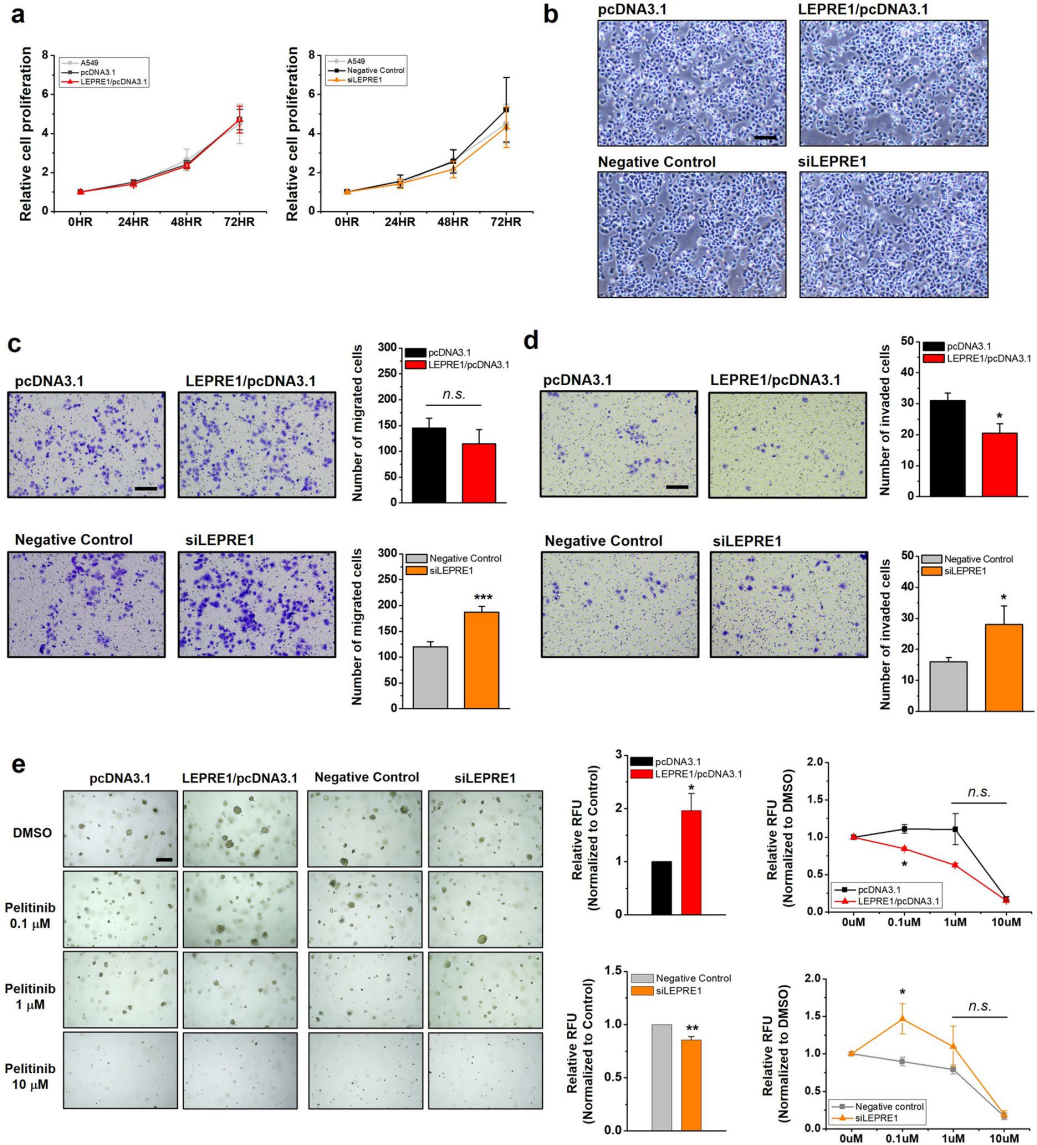
LEPRE1 affects cell morphology, migration/invasion, and colonization in lung cancer-derived A549 cells. **(a**) Comparison of cell growth in parental or transfected (pcDNA3.1, LEPRE1/pcDNA3.1, Negative Control siRNA, or siLEPRE1) A549 cells. Equal numbers of cells were cultured in 96-well plates for 72 h. After the indicated time points, proliferation rates were analyzed using WST-1 proliferation assays. Error bars represent the SEM from three independent experimental replicates. (**b**) Comparison of cell morphology in transfected A549 cells (pcDNA3.1, LEPRE11/pcDNA3.1, Negative Control siRNA, or siLEPRE1). Scale bar, 800 μm. (**c**,**d**) Migration (**c**) and invasion (**d**) abilities of transfected A549 cells were evaluated using Transwell or Matrigel-coated Transwell plates, respectively. After 24 h, the migrated or invading cells were fixed, stained with crystal violet, and photographed. Representative images of Transwell inserts demonstrating stained migrating or invading cells are shown (left side). The relative migration or invasive abilities of the indicated cells are displayed in the bottom panel. Values shown are means ± SEMs from three independent experiments. Scale bar, 200 μm; *n.s.* not significant; *p < 0.05; ***p < 0.001. (**e**) Colony formation assays of transfected A549 cells treated with DMSO or the indicated concentration of pelitinib for 7 days. Representative images of colonies are shown (left side). The ability of transfected A549 cells to form colonies was normalized to the control cells, which are shown in the middle negative control column. Responsiveness to pelitinib treatments was normalized to each corresponding DMSO control (right side). Scale bar, 200 μm. Data are presented as the mean ± SEM of three independent experiments. *n.s.* not significant; *p < 0.05; **p < 0.01.

To further investigate the role of LEPRE1 in pelitinib responsiveness, we evaluated the cellular morphology and migration of A549 cells. Cell proliferation was comparable among the parental, LEPRE1-overexpression, vector control cells, and LEPRE1-knockdown and negative control cells (Fig. 5a). In terms of cell morphology, LEPRE1-overexpression cells were more likely to adhere to each other and typically formed cell clusters compared to vector control cells (Fig. 5b, upper images). In contrast, LEPRE1-knockdown A549 cells displayed a mesenchymal phenotype, including decreased cell–cell contacts and increased pseudopodia formation (Fig. 5b, lower images). These characteristics are consistent with the typical cellular structure of EMT.

EMT is a physiological process used by tumor cells to acquire critical oncogenic features, such as migration/invasion, stemness, and drug resistance^29^. Therefore, we then examined whether LEPRE1 levels affected cell migration, invasion, and colony formation. The rate of cell growth was not affected by LEPRE1 (Fig. 5b). Accordingly, we concluded that the differences observed in the migration experiments were mainly due to the influence of LEPRE1 on cell motility. The results showed that LEPRE1-overexpression suppressed the migration and invasion ability of A549 cells, while LEPRE1-knockdown promoted migration and invasion (Fig. 5c,d). Based on colony formation assays, ectopic LEPRE1 expression promoted, and LEPRE1 knockdown suppressed colony formation in soft agar (Fig. 5e). Considering these results, we suggest that LEPRE1 modulated the cell fate of clonogenic properties or EMT by regulating cell morphology and migration/invasion.

Moreover, we further investigated the growth of each clone in the presence of pelitinib (0, 0.1, 1, and 10 μM) using clonogenic survival assays. Pelitinib treatment significantly reduced the number of colonies in a dose-dependent manner. We also observed that the overexpression of LEPRE1 resulted in greater pelitinib sensitivity. In contrast, LEPRE1-knockdown A549 cells exhibited greater pelitinib resistance compared with negative control cells.

### LEPRE1 regulates EMT/mesenchymal-epithelial transition (MET) state via EGFR-independent signaling pathways

**Figure 6:**
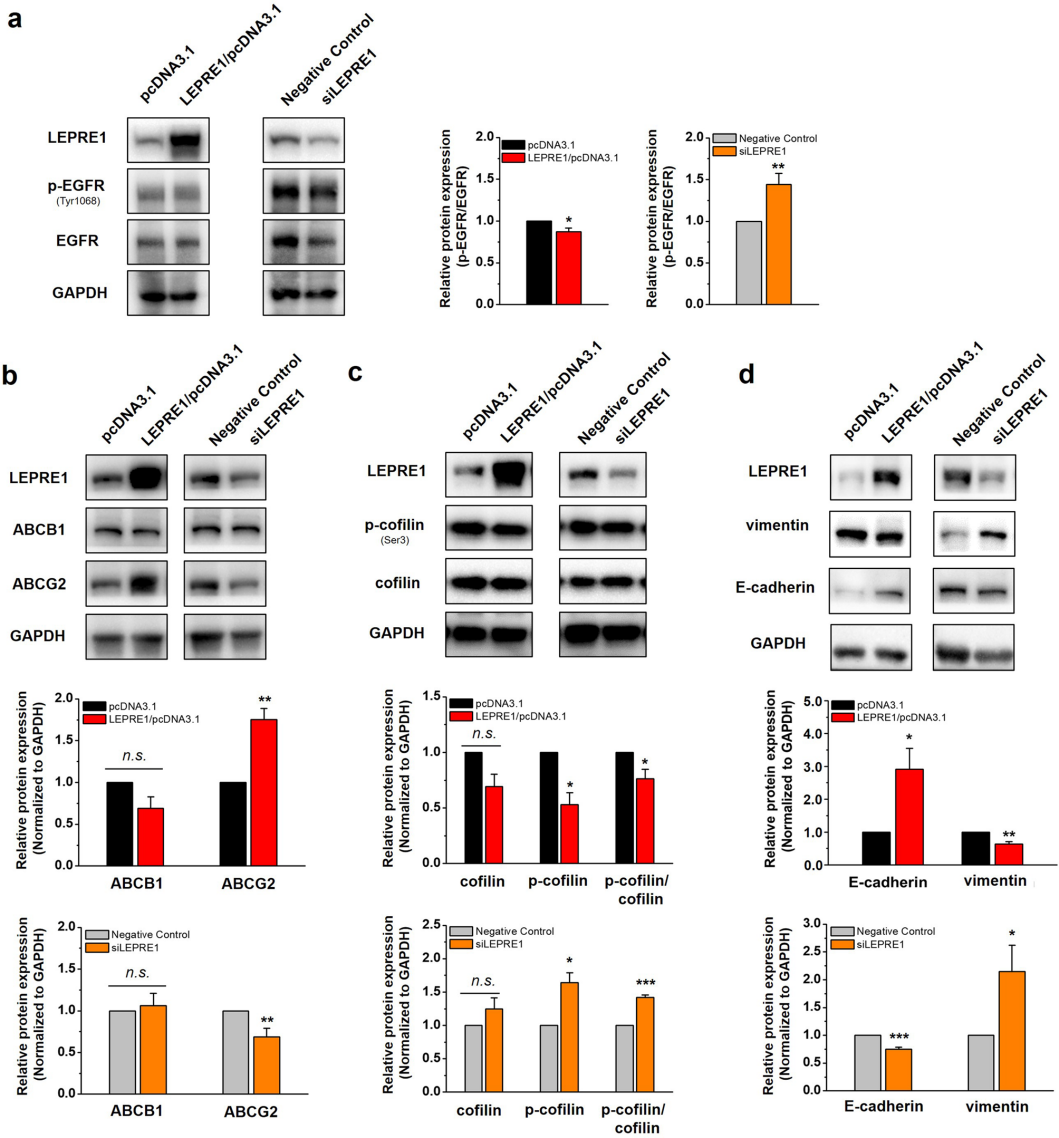
LEPRE1 affects the expression of EMT and MET markers in lung cancer-derived A549 cells. **(a**– **d**) Western blot analysis of EGFR and p-EGFR (**a**), ABCB1 and ABCG2 (**b**), p-cofilin and cofilin (**c**), and vimentin and E-cadherin (**d**) in transfected A549 cells. The relative expression levels of p-EGFR/EGFR (**a**), ABCB1 and ABCG2 (**b**), p-cofilin/cofilin (**c**), and vimentin and E-cadherin **(d)** were normalized to GAPDH and are presented in the lower panel. GAPDH protein was used as an internal control. The data are presented as the mean ± SEM of three independent experiments. *n.s.* not significant; *p < 0.05; **p < 0.01; ***p < 0.001. All western blots are pre-cut. Membranes were often stripped and reprobed for multiple antibodies.

To further investigate the mechanism of LEPRE1 affecting pelitinib responsiveness in A549 cells, we focused on the EGFR signaling pathway, multidrug resistance (MDR), actin rearrangement, and EMT. In LEPRE1-knockdown cells, EGFR and p-EGFR expression were decreased compared with those in the negative control cells. However, the p-EGFR/EGFR ratio was increased in LEPRE1-knockdown and decreased in LEPRE1-overexpressing A549 cells (Fig. 6a).

To determine the cause of drug sensitivity/resistance according to the level of LEPRE1 expression, we evaluated the expression of ABC transporters ABCB1 and ABCG2. ABCG2 expression levels in LEPRE1-knockdown cells were lower than those in the negative control cells (Fig. 6b). In contrast, ABCG2 expression was increased in LEPRE1-overexpressing A549 cells (Fig. 6b).

According to a previous study, cofilin expression is increased in primary fibroblasts of osteogenesis imperfecta patients with mutations in the LEPRE1 gene^30^. To evaluate cofilin expression in transfected A549 cells, we performed western blotting for cofilin and p-cofilin. The levels of cofilin and p-cofilin and the p-cofilin/cofilin ratios were increased in LEPRE1-knockdown cells compared with those in the negative controls. In contrast, the expression of cofilin and p-cofilin and the p-cofilin/cofilin ratio were decreased in LEPRE1-overexpressing A549 cells (Fig. 6c).

To determine whether LEPRE1 induced EMT in A549 cells, we performed western blot analysis to evaluate the levels of EMT-related proteins, including vimentin and E-cadherin. As shown in Fig. 6d, E-cadherin levels were decreased, and vimentin increased when LEPRE1 was knocked down. In contrast, increased E-cadherin levels and decreased vimentin levels were observed in LEPRE1-overexpressing cells. These results indicated that LEPRE1 might be involved in EMT/MET regulation and regulate the tumor state via either EGFR-dependent or EGFR-independent pathways.

EMT is a crucial step of metastasis, it is therefore essential to determine whether LEPRE1 expression is associated with the clinical outcome of cancer patients. We performed Kaplan–Meier survival analyses on lung squamous cell carcinoma (LUSC) from human protein atlas (https://www.proteinatlas.org/, Supplementary Fig. 4), and found low expression of LEPRE1 to be consistently associated with poor clinical outcomes during the early stages of cancer; meanwhile, LEPRE1 overexpression is associated with poor clinical outcomes during the late stages of cancer. This suggests LEPRE1 as a novel therapeutic target against cancer metastasis at various cancer stages.

### LEPRE1 induces pelitinib-specific drug responsiveness by regulating ABCG2 expression

**Figure 7:**
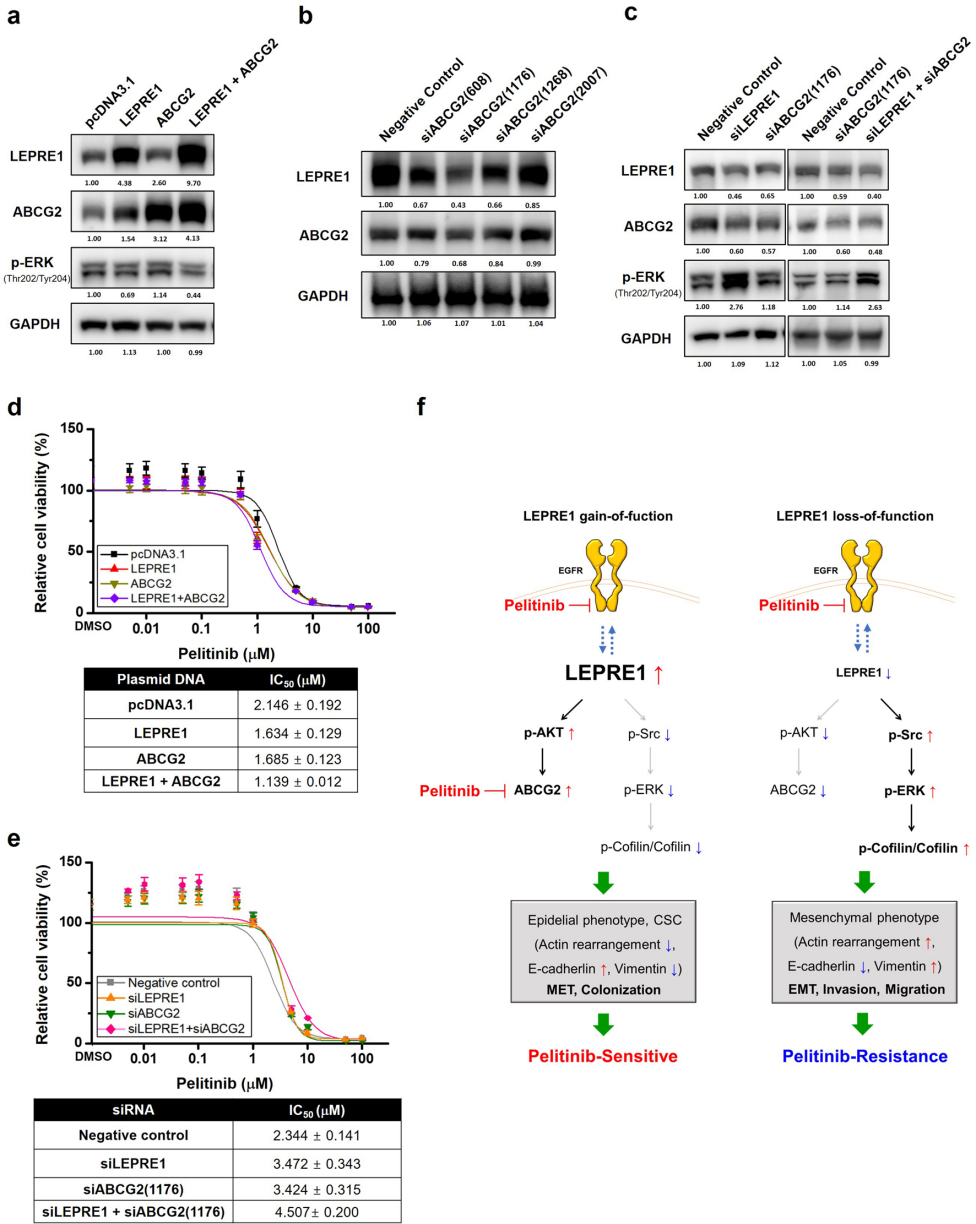
Proposed mechanism of action for LEPRE1 in pelitinib-sensitive or pelitinib-resistant A549 cells. **(a)** Western blot analysis of LEPRE1, ABCG2 and, p-ERK expression after overexpression or co-transfection of LEPRE1 and ABCG2 in the A549 cell lien. (**b)** Protein expression levels of ABCG2 determined following A549 cells being transfected with ABCG2-si608, -si1176, -si1268, -si2007, or negative control siRNA. (**c)** Western blot analysis of LEPRE1, ABCG2, and p-ERK expression after siRNA single or co-transfection of siLEPRE1 and siABCG2 in A549 cells. All western blots are pre-cut. Membranes were often stripped and reprobed for multiple antibodies. (**d,e**) LEPRE1-, ABCG2-overexpression or co-transfection **(d)** or LEPRE1, ABCG2 knockdown or double knockdown **(e)** A549 cells were treated with pelitinib for 3 days. Cell viability was determined using a WST-1 proliferation assay. (**f)** ABCG2 Schematic model showing that overexpression of LEPRE1 induces AKT activation and ABCG2 overexpression, which results in increased E-cadherins levels and a colonization phenotype, ultimately leading to changes resulting in sensitivity to pelitinib. Meanwhile, silencing of LEPRE1 results in activation of the Src/ERK/Cofilin signaling pathway, which leads to increased vimentin levels and the acquisition of an EMT-like phenotype. This ultimately leads to changes resulting in resistance to pelitinib.

To confirm the relationship between LEPRE1 and ABCG2 role in pelitinib-induced responsiveness, LEPRE1 or ABCG2 plasmid was transfected with single or co-transfection (Fig. 7a). In ABCG2-overexpressing A549 cell, the LEPRE1 expression was increased, along with a slight increase in the p-ERK levels, compared to pcDNA3.1 vector control. The reduced p-ERK expression was detected in LEPRE1 and ABCG2 co-transfected cell, and was more reduced compared to LEPRE1-overexpressing cells.

To examine the role of LEPRE1 and ABCG2 in pelitinib cytotoxicity, transfected cells were treated with pelitinib (Fig. 7a). Pelitinib produced the most significant inhibitory effect on LEPRE1 + ABCG2 co-transfected cells, with an IC_50_ value of 1.139 μM compared to A549_pcDNA3.1 cells. In LEPRE1- or ABCG2-expressing cells, pelitinib inhibited proliferation, although with less potency than co-transfected cells (Fig. 7d).

Then, we investigated the efficacy of siRNAs against endogenous ABCG2 in A549 cells. Based on quantitative immunoblotting results, si1176 suppressed ABCG2 expression to 67% of the negative control (Fig. 7b). To assess the knockdown effect of ABCG2 in pelitinib induced drug sensitivity, we transfected siRNA against ABCG2 and LEPRE1 single or co-transfection (Fig. 7c). In ABCG2 and LEPRE1 double knockdown cells, the p-ERK density was increased along with the IC_50_ value compared to negative control cells. The siABCG2 single transfected cells have similar IC_50_ value to that of A549_siLEPRE1 cells following pelitinib treatment (Fig. 7e).

These results suggest that LEPRE1 and ABCG2 regulate pelitinib responsiveness by regulating p-ERK expression.

## Discussion

In this study, we performed a bioinformatics meta-analysis based on the GDSC and COSMIC databases, focusing on EGFR signaling pathway drugs, including pelitinib. We found that increased LEPRE1 expression was related to pelitinib sensitivity. In-silico result-based predictions were experimentally validated in AML and A549 cell lines using drug response and mechanism studies. Notably, we first report LEPRE1 as a putative biomarker for the selection of pelitinib to treat AML and lung cancer.

LEPRE1 is associated with type VIII osteogenesis imperfecta and has been extensively investigated because of its protective function in cell homeostasis and its correlation with osteogenesis imperfecta^31^. Interestingly, cancer is rarely concurrently seen in patients with osteogenesis imperfecta^32^, and it has been suggested that these patients experience some cancer protection; however, there is currently no direct evidence to support this hypothesis^33^. It has also been reported that an increase in EGFR induces a decrease in LEPRE1 expression in breast cancer^34^. Recently, the possibility of direct binding between EGFR and LEPRE1 has been suggested^35^. We confirmed the binding of LEPRE1 to EGFR using immunoprecipitation experiments (data not shown). Thus, a mechanistic molecular relationship exists between EGFR and LEPRE1 in tumor development.

Pelitinib (EKB-569) is an irreversible EGFR-TKI that covalently binds and inhibits EGFR^23^. Interestingly, pelitinib decreases p-AKT and p-ERK protein levels in hepatocellular carcinoma, where EGFR-targeted therapy is ineffective^22^. These reports indicate that pelitinib has various targets and can exert its drug activity in an EGFR-independent manner. Thus, pelitinib drug responsiveness relative to LEPRE1 expression levels was effective in AML (EGFR-low) and A549 (EGFR-high) cell lines.

We also found that high LEPRE1 expression in A549 cells induced AKT activation, the overexpression of ABCG2 and E-cadherin, and cell colonization. In LEPRE1-overexpressing cells, EGFR expression was increased, but no significant change was observed in the p-EGFR/EGFR ratio compared with control cells. Endogenous LEPRE1 in A549 cells was expressed in the ER, peri-nucleus regions, and vesicles (data not shown). It has also been previously reported that membrane protein trafficking is regulated by PI3K/AKT signaling^36^. Accordingly, we speculated that LEPRE1 might regulate the trafficking of membrane proteins EGFR and ABCG2 via the AKT-dependent signaling pathway. In many kinds of human tumor cells, ABCG2 expression on the plasma membrane contributes to MDR during chemotherapy, and ABCG2 is also known to be a marker for identifying cancer stem cells (CSCs) in lung cancers^37^. According to Wang et al*.*^38^, ABCG2 expression and AKT activation were positively associated with CSC-like phenotypes. Increased ABCG2 expression causes an increase in E-cadherin, inhibits cell migration, induces MET progression, and promotes higher lung metastatic colonization^37^. However, the interactions between LEPRE1 and EGFR/AKT/ABCG2 signaling concerning CSC-like phenotypes remain elusive and require further clarification.

Pelitinib has been shown to exhibit high-affinity interaction with the ABCG2 transporter, which acts as a competitive inhibitor of ABCB1/ABCG2 to selectively kill CSC-like cells after hyperthermia in lung cancer^27^. According to the “cancer stem cell” hypothesis, these drug-resistant CSCs are responsible for driving tumor re-growth, and ABCG2 is probably a pivotal efflux transporter that contributes to preserving the CSC sanctuary under chemotherapeutic pressure^39^. In ABCG2-expressing tissues where local drug concentrations are high, the drugs can inhibit ABCG2 function and promote intracellular accumulation and cytotoxic activity, thereby reversing MDR^40^, supporting the hypothesis that the reversing effect of pelitinib in LEPRE1-overexpressing cells is due to increased cytotoxicity caused by ABCG2 overexpression. Our results showed that A549_LEPRE1 + ABCG2 cells were more sensitive to pelitinib than LEPRE1 or ABCG2 expressing cells. Meanwhile, A549_siLEPRE1 + siABCG2 cells were more resistance to pelitinib then siLEPRE1 or siABCG2 transfected cells. This suggests that LEPRE1 may regulate ABCG2 expression while pelitinib enhances accumulation, thereby restoring cellular sensitivity toward ABCG2 substrate cytotoxic drugs by directly blocking ABCG2 function.

LEPRE1 silencing reduced EGFR expression in A549 cells but increased the p-EGFR/EGFR ratio. According to Liu et al*.*^12^, receptor tyrosine kinases (RTKs) and the abnormal activation of downstream signaling molecules have compensatory functions against the inhibition of EGFR by triggering the PI3K/AKT and MAPK signaling cascades. Activation of bypass mechanisms of resistance to RTK inhibition via other RTKs or alternative downstream signaling molecules also contributes to acquired resistance^41^. From these pieces of evidence, we speculated that the activation signals by the EGFR-independent pathway induced Src and ERK phosphorylation, ultimately affecting the p-cofilin/cofilin ratio and vimentin overexpression.

In addition, knockdown of LEPRE1 in cells resulted in an EMT-like phenotype, and migration and invasion were increased. According to previous reports, Src mediates the migration and invasion of breast cancer and hepatocellular carcinoma cells through the downstream activation of the ERK pathway^42–44^. It is also known that Src activation causes actin depolymerization via the MEK/ERK/Cofilin cascade, inducing tumor transformation^45^. Oncogenic transformation is accompanied by morphological changes due to changes in actin dynamics and adhesion activity. Furthermore, cofilin-1 has critical roles in switching from EMT and promoting cell migration and invasion by regulating actin cytoskeleton organization^46^. In addition, an altered cytoskeleton and nuclear lamina organization occur in fibroblast cells of patients with osteogenesis imperfecta carrying mutations in LEPRE1^30^. Thus, LEPRE1 silencing induces EMT through actin reconstitution via a series of Src/ERK/Cofilin cascades.

Due to morphological and physiological changes found in EMT-induced cells, the effectiveness of EGFR-TKIs is limited by acquired resistance, which represents the classical paradigm of molecular-targeted therapies in NSCLC. Recently, a growing number of studies have suggested a relationship between EMT and drug resistance. Along with the decreased expression of E-cadherin, vimentin expression is elevated, illustrating the emergence of EMT^47^. For example, a patient with lung cancer who developed acquired resistance to erlotinib exhibited EMT properties in the tissue sample. In addition, a gefitinib-resistant subline of HCC827 cells shows phenotypic and molecular changes that are consistent with EMT^48^. Although the mechanisms that regulate EMT are non-linear complex networks^49^, we uncovered one mechanism of EMT induction and drug resistance due to reduced LEPRE1 levels (Fig. 7).

Based on our results, we can suggest a method of administering drugs in which EGFR-TKIs resistance caused by LEPRE1-AKT-ABCG2 overexpression in CFC-like cells can be overcome with pelitinib treatment. Furthermore, maximum drug effects can be expected when pelitinib and other drugs are administered in combination. It may also be possible to overcome pelitinib resistance caused by EMT induction in the absence of LEPRE1 by inhibiting Src or MECK signaling. Blockage of the MAPK pathway is possible using MEK inhibitors^50^, including trametinib (GSK1120212), which is a MEK1/2 inhibitor with a longer half-life than previous MEK inhibitors^51,52^. In addition, a synergistic effect can be induced by co-administering pelitinib and various other anti-cancer drugs to advanced cancer patients that express high levels of LEPRE1 and ABCG2. This finding highlights the possible clinical benefit of combining targeted EGFR inhibitor-based therapies with ABCG2 substrate conventional chemotherapeutic drugs.

In conclusion, we verified the expression of LEPRE1 as a candidate biomarker and the reactivity of the EGFR-TKI drug pelitinib using in vitro experiments executed about computed values derived from bioinformatics. Through this process, we confirmed the reliability of the in-silico prediction results. In addition, we provided a function of LEPRE1 as a new biomarker and its novel mechanism for EGFR-TKI drug responsiveness in various AML-derived and lung cancer-derived cells. Indeed, we found no direct association between LEPRE1 and EGFR in the drug reactivity of pelitinib. However, we find it encouraging that the values calculated by the artificial intelligence computer can consider complex internal molecular pathways rather than predict simple target-drug pairings. There is a need to derive many new biomarkers for the development of customized anticancer drugs and accurate cancer diagnosis, which can be advanced based on in-silico analysis. In addition, it is necessary to investigate the molecular mechanisms of EMT, MET, and tumor metastasis and determine the reaction mechanism of related drugs.

## Methods

### In-silico analysis

See Supplementary methods for details.

### Cell culture and kinase inhibitors

Human AML cell lines THP-1, U937, KG-1, and HL60 were obtained from the Korean Cell Line Bank (Seoul National University, Seoul, Korea), and the lung cancer cell line A549 was obtained from American Type Culture Collection (ATCC, Manassas, VA, USA). All cells were maintained in RPMI-1640 medium (HyClone; GE Healthcare Life Sciences, Logan, UT, USA) supplemented with 10% fetal bovine serum (FBS; Gibco, Thermo Fisher Scientific, Grand Island, NY, USA) and penicillin–streptomycin. The EGFR-TKIs afatinib, erlotinib, gefitinib, lapatinib, and pelitinib were obtained from Selleck Chemicals. Stock solutions of all drugs were prepared in dimethyl sulfoxide (DMSO; Sigma-Aldrich, St. Louis, MO, USA) and stored at − 80 °C.

### Plasmid constructs and small interfering RNAs (siRNAs)

See Supplementary methods for details.

### Electroporation and transfection

THP-1 and KG-1 cells were washed with serum-free RPMI-1640 medium before transfection. Plasmid (4 μg) or siRNA (100 μg) was mixed with 1 × 10^6^ THP-1 or KG-1 cells suspended in serum-free RPMI-1640 in a 2 mm electroporation cuvette and electroporated in a BTX ECM 830 square wave pulse generator (Harvard Apparatus, Holliston, MA, USA) under the following conditions: choose mode, LV; pulse length, 10 ms; charging voltage, 150 V. The electroporated cells were transferred to culture flasks containing RPMI-1640 plus 20% FBS and incubated for 24 h. The viable cells were then counted using Trypan blue staining and seeded into plates at desired densities, which were treated with pelitinib for the next 24 h. A549 cells were transfected using Lipofectamine 2000 (Invitrogen; Thermo Fisher Scientific, Inc., San Diego, CA, USA), according to the manufacturer's instructions.

### Western blotting and antibodies

See Supplementary methods for details.

### Detection of protein phosphorylation

See Supplementary methods for details.

### Cytotoxicity assessment

Cytotoxicity was determined using a colorimetric assay using Cell Proliferation Reagent WST-1 (Roche Applied Science, Indianapolis, IN, USA). The assay was performed in 96-well plates seeded with approximately 10,000 cells/well. After 72 h of transfection, 10 μL of the WST-1 solution was added to the culture medium and incubated for 1 h at 37 °C. Absorbance was subsequently measured using an ELISA SpectraMax iD3 Microplate Reader (Molecular Devices, LLC, Sunnyvale, CA, USA) and analyzed using SoftMax Pro 7.1 software, applying wavelengths 450 nm for measurements and 650 nm as reference. Cell proliferation was calculated by comparing absorbance values of the samples after background subtraction^24^. The IC_50_ values were determined by fitting curves using non-linear least-squares regression in a sigmoidal dose–response model with a variable slope using Origin 6.1 software (OriginLab Corporation, Northampton, MA, USA). Each assay was performed in triplicate for each drug concentration, and assays were independently repeated thrice.

### Proliferation assays

Cellular growth curves were plotted using cellular viability values assessed using the WST-1 method. Cells were seeded into 96-well plates at a density of 3 × 10^3^ cells/well in 100 μL of culture medium. At various time points (0, 24, 48, and 72 h) after seeding, the cells in each well were treated with 10 μL of the WST-1 solution for 1 h at 37 °C. Absorbance was subsequently determined using a microplate ELISA SpectraMax iD3 Microplate Reader (Molecular Devices) and analyzed using SoftMax Pro 7.1 software. Wavelengths of 450 nm were used for measurements and 650 nm as reference.

### Transwell migration and invasion assays

Transwell migration and invasion assays were performed using 24-well Transwell (8-μm pore size; Corning Life Sciences, Bedford, MA, USA). For migration assays, 5 × 10^4^ cells were plated into the upper insert containing 0.1% FBS culture medium with the lower chamber containing 10% FBS culture medium and incubated at 37 °C under 5% CO_2_ for 24 h. Cells that migrated through the membrane were fixed and stained with crystal violet (Sigma-Aldrich). Invasion assays were performed in 24-well Matrigel (Corning Life Sciences) coated chambers. Again, 5 × 10^4^ cells were plated into the upper insert containing 0.1% FBS culture medium with the lower chamber containing 10% FBS culture medium and incubated for 24 h. Cells that invaded through the Matrigel were fixed and stained with crystal violet. The cells were counted in four randomly selected microscopic visual fields. Each sample was assayed in triplicate, and three independent assays were performed. The mean ± SEM was calculated for each sample.

### Soft agar colony-forming assays

Colony formation was assessed using the CytoSelect 96-Well Cell Transformation Assays kit (Cell Biolabs, Inc., San Diego, CA, USA) according to the manufacturer’s protocol. Briefly, a base agar layer of 0.6% agar, Dulbecco’s modified Eagle’s medium, and 10% FBS was poured into each well and covered with a cell agar layer of the same composition containing a density of 3 × 10^3^ cells/well plus pelitinib at various concentrations (0, 0.1, 1, and 10 μM). After 7 days, the agar was solubilized, and the mixture was incubated with a CyQuant working solution. Fluorescence was measured at 485/520 nm using a 96-well microplate reader. Growth inhibition rates were calculated relative to untreated control cultures.

### Statistical analysis

Values are presented as mean ± SEM of experiments conducted with replicates as indicated (n). Data were analyzed using Origin 6.1 software (OriginLab, Northampton, MA, USA) and/or Microsoft Excel. Differences between control and treated groups were analyzed using one-way analysis of variance (ANOVA) followed by a post-hoc Dunnett’s pairwise comparison or linear trend test to determine a dose-dependent effect.

## Supplementary Information

Supplementary Information.

## Data Availability

All data generated and analyzed during the current study are available from the corresponding author on reasonable request.
