# Supplementary material for: Biomarker LEPRE1 induces pelitinib-specific drug responsiveness by regulating ABCG2 expression and tumor transition states in human leukemia and lung cancer
Source: Sci Rep. 2022 Feb 21;12:2928. doi: 10.1038/s41598-022-06621-w (PMC8861100; doi:10.1038/s41598-022-06621-w)
Supplement: Supplementary file 1 — Supplementary Information. [file 41598_2022_6621_MOESM1_ESM.pdf]

Supplementary Information

Biomarker LEPRE1 induces pelitinib-specific drug responsiveness by regulating ABCG2 expression and tumor transition states in human leukemia and lung cancer

A-Ram Lee[1,3], Sunho Lee[2,4], Jee Yoon Shin[2], Ji-Young Kim[1], Kyoung-Sik Moon[1,*] and Joungsun Jung [2,*]

[1]Department of Advanced Toxicology, Korea Institute of Toxicology (KIT), Daejeon, 34114, Republic of Korea

[2]Genome Data Integration Centre, Syntekabio Inc., Daejeon, 34025, Republic of Korea

[3]Present address: Bio Center, LegoChem Biosciences, Inc., Daejeon, 34002, Republic of Korea

[4]Present address: Department of Computer Science and Engineering, Seoul National University, Seoul, 08826, Republic of Korea

Supplementary Methods

In silico analysis

Plasmid constructs and small interfering RNAs (siRNAs)

Western blotting

Detection of protein phosphorylation

Supplementary Figures

Supplementary Figure 1. Knockdown and overexpression of LEPRE1 on KG-1 and THP-1 AML cells.

Supplementary Figure 2. Effect of EGFR-TKIs on KG-1 and THP-1 AML cells.

Supplementary Figure 3. Effect of EGFR-TKIs on A549_LEPRE1 and A549_siLEPRE1 cells

Supplementary Figure 4. Survival analysis of LEPRE1 in LUSC patients.

Full-length blots for cropped images

Supplementary Figure 5. Full-length blots of western blot figures shown in Figure 1. c, d.

Supplementary Figure 6. Full-length blots of western blot figures shown in Figure 2. b, d, e,f,g

Supplementary Figure 7. Full-length blots of western blot figures shown in Figure 3. c, d.

Supplementary Figure 8. Full-length blots of western blot figures shown in Figure 4. b, c, d.

Supplementary Figure 9. Full-length blots of western blot figures shown in Figure 6. a, b, c, d.

Supplementary Figure 10. Full-length blots of western blot figures shown in Figure 7. a, b, c.

Supplementary Figure 11. Full-length blots of western blot figures shown in Supplementary Figure 2.

Supplementary Methods

In silico analysis

To select candidate genes associated with drug responsiveness to EGFR-TKIs in blood cancer cells, we used the GDSC1 dataset consisting of 987 cell lines and 367 compounds. Of these, 167 blood cancer cell lines, including acute lymphoblastic leukemia (ALL, n = 37), acute myeloid leukemia (AML, n = 25), chronic myeloid leukemia (CML, n = 5), and diffuse large B-cell lymphoma (DLBC n = 100) were analyzed using gene expression data, and six EGFR signaling pathway drugs (afatinib, cetuximab, erlotinib, gefitinib, lapatinib, and pelitinib) were analyzed. Drug response data and gene expression data were downloaded from GDSC (https://www.cancerrxgene.org) and the Catalogue of Somatic Mutations in Cancer (COSMIC) database (https://cancer.sanger.ac.uk/cosmic), respectively. Among all combinations of the six drugs and approximately 15,000 genes, we first collected the combinations with a significant correlation between drug responsiveness and gene expression level using Spearman's correlation test (p threshold < 0.01). For each combination collected (drug X and gene Y), we then used a t-test to further investigate the significance of drug responsiveness according to gene expression level and designated combinations with p-value < 0.01 as candidates. Specifically, the blood cancer cells were divided into two groups according to gene Y expression levels. The Dy set of cells were those with a gene expression z-score less than zero, and the remaining cells were the Uy set. Statistical significance between the drug responses of Dy and Uy was calculated by t-test analysis using the SciPy.stats library in Python. The candidates were then re-evaluated as described below.

For each candidate drug/gene pair, we calculated the difference in drug responsiveness between the two groups marked by COSMIC[1] as the gene being overexpressed (z-score > 2) and those being under-expressed (z-score < −2). The analysis was repeated to avoid bias due to limitations in the number of differentially expressed samples, applying the lower criteria of overexpressed and under-expressed of z-score > 1 and z-score < −1, respectively. We denoted the average value of the two differences as the half-maximal inhibitory concentration (IC_{50}). The candidates were then sorted in descending order according to the IC_{50} effect.

Plasmid constructs and small interfering RNAs (siRNAs)

Human cDNAs encoding full-length LEPRE1 and full-length ABCG2 subcloned in-frame into pcDNA3.1(+) were purchased from Genscript (Piscataway, NJ, USA). The siRNAs were specific for LEPRE1 (siLEPRE1-1010, 5′-CCU CCC AUC GCA UUA UAA UTT-3′; siLEPRE1-1842, 5′-GCA GAG AGG AAG GAU GAU ATT-3′; siLEPRE1-2293, 5′-GCA GUG AAU CGA AGC CCA ATT-3′).

The siRNAs were specific for ABCG2 (siABCG2-608, 5’-CGA CCU GCC AAU UUC AAA UTT-3’, 5’-AUU UGA AAU UGG CAG GUC GTT-3’; siABCG2-1176, 5’-CCA CUG CUG UGG CAU UAA ATT-3’, 5’-UUU AAU GCC ACA GCA GUG GTT-3’; siABCG2-1268 5’-GCG GAG AUU UAU GUC AAC UTT-3’, 5’-AGU UGA CAU AAA UCU CCG CTT-3’; siABCG2-2007, 5’-CGG CUU UGC AGC AUA AUG ATT-3’, 5’-UCA UUA UGC UGC AAA GCC GTT-3’). The negative control siRNA (NC, 5′-UUC UCC GAA CGU GUC ACG UTT-3′) was obtained from GenePharma (Shanghai, China).

Western blotting and antibodies

Cells were treated with increasing concentrations of EGFR-TKIs (0.1–10 μM) for 24 h at 37 °C. The cells were then washed with ice-cold phosphate-buffered saline (PBS) and lysed in PRO-PREPTM Protein Extraction Solution (iNtRON Biotechnology, iNtRON Biotechnology, Seongnam, Korea). Protein concentration of the cell lysates was determined using Bradford Reagent (Biorad Laboratories, Hercules, CA, USA), and equal amounts of protein were subjected to SDS-PAGE. The separated proteins were transferred to polyvinylidene difluoride (PVDF) membranes (Amersham Biosciences, Buckinghamshire, UK) and blocked with bovine serum albumin (EzBlock BSA, Atto Corporation, Tokyo, Japan) in PBS for 30 min at room temperature before incubation overnight at 4 °C with primary antibodies. The following commercially available antibodies were used. A rabbit monoclonal antibody directed against a synthetic peptide within human LEPRE1 was purchased from Abcam (ab154799, Abcam, Cambridge, MA, USA). Rabbit anti-poly (ADP-ribose) polymerase (PARP, #9542), ATP-binding cassette subfamily C member 1 (ABCC1, #72202), ABCG2 (#4477), cofilin (#5175), and GAPDH (#2118) antibodies were obtained from Cell Signaling Technology (Danvers, MA, USA). Mouse monoclonal antibodies specific for human EGFR (#373746), E-cadherin (#8426), vimentin (#6260), and β-actin (#47778) were obtained from Santa Cruz Biotechnology (Santa Cruz, CA, USA). The PVDF membranes were washed with Tris-buffered saline containing 0.05% Tween 20 and incubated for 1 h at room temperature with horseradish peroxidase-conjugated goat anti-rabbit or goat anti-mouse antibodies as appropriate (Jackson ImmunoResearch Laboratories, West Grove, PA, USA). Immune complexes were detected using chemiluminescence reagents (Thermo Fisher Scientific, Grand Island, NY, USA).

Detection of protein phosphorylation

To examine EGF-stimulated phosphorylation of proteins, cells were seeded in serum-free medium into 6-well plates and allowed to adhere. The adhered cells were pre-treated with pelitinib at various concentrations (0, 0.1, 1, and 10 μM) for 5 h before exposure to 20 ng/mL recombinant human EGF (hEGF; R&D Systems, Minneapolis, MN, USA) for 15 min. Cells were washed and scraped in lysis buffer (PRO-PREPTM Protein Extraction Solution, iNtRON Biotechnology) containing various phosphatase and protease inhibitors. The extracted proteins (20 μg) were analyzed using western blotting. The blots were probed with p-EGFR (Tyr1068, #3777), p-AKT (Ser473, #9271), p-MEK1/2 (Ser217/221, #9154), p-Src (Tyr416, #2101), p-ERK1/2 (Thr202/Tyr204, #4370), and p-cofilin (Ser3, #3313) antibodies (Cell Signaling Technology). GAPDH was immunostained as a sample loading control.

Reference

1. Yang, W. *et al*. Genomics of Drug Sensitivity in Cancer (GDSC): a resource for therapeutic biomarker discovery in cancer cells. *Nucleic Acids Res.* **41**, D955–D961 (2013).

Supplementary Figures

Supplementary Figure 1

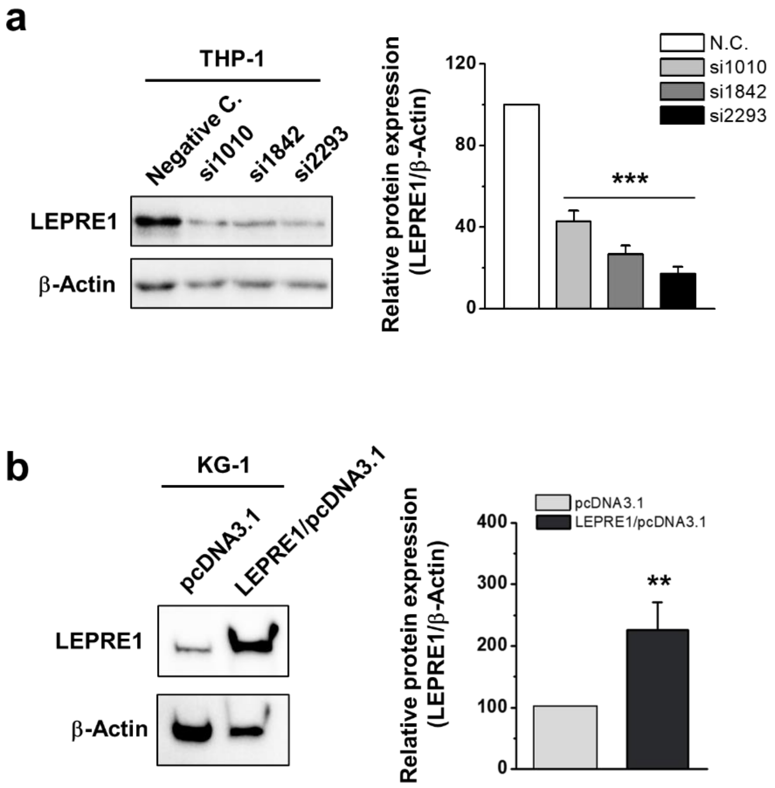

Supplementary Figure 1. Knockdown and overexpression of LEPRE1 on KG-1 and THP-1 AML cells. **(a)** Protein expression levels of LEPRE1 determined following THP-1 cells being electroporated with LEPRE1-si1010, LEPRE1-1842, LEPRE1-2293, or negative control siRNA. The bands were quantified and are presented as the mean ± SEM of three independent experiments. ***p < 0.001. (**b**) Western blot analysis of LEPRE1 expression after overexpression of LEPRE1 in the AML-derived KG-1 cell line. The bands were quantified and are presented as the mean ± SEM of three independent experiments. **p < 0.01.

Supplementary Figure 2

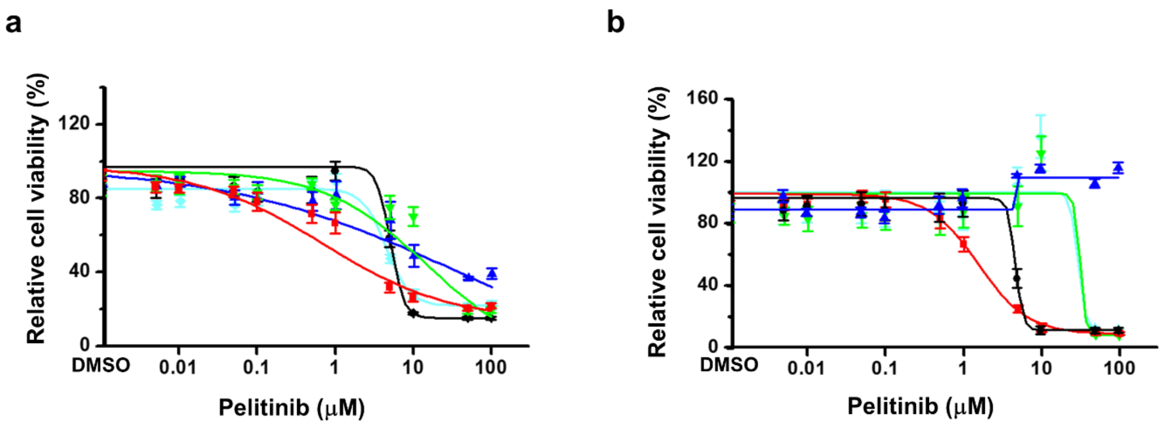

IC_{50} (μM)

EGFR-TKIs	KG-1	THP-1
Pelitnib	0.682 ± 0.181	1.531 ± 0.174
Afatinib	5.102 ± 0.219	4.762 ± 1.011
Erlotinib	> 10	> 10
Gefitinib	> 10	> 10
Lapatinib	4.384 ± 0.316	> 10

Supplementary Figure 2. Effect of EGFR-TKIs on KG-1 and THP-1 AML cells.

(**a**, **b**) KG-1 cells (**a**) and THP-1 cells (**b**) and incubated with EGFR-TKIs (afatinib, erlotinib, gefitinib, lapatinib, and pelitinib) for 72 h. Cell viability was determined using WST-1 proliferation assays. The IC_{50} value for each condition is presented in the lower panel.

Supplementary Figure 3

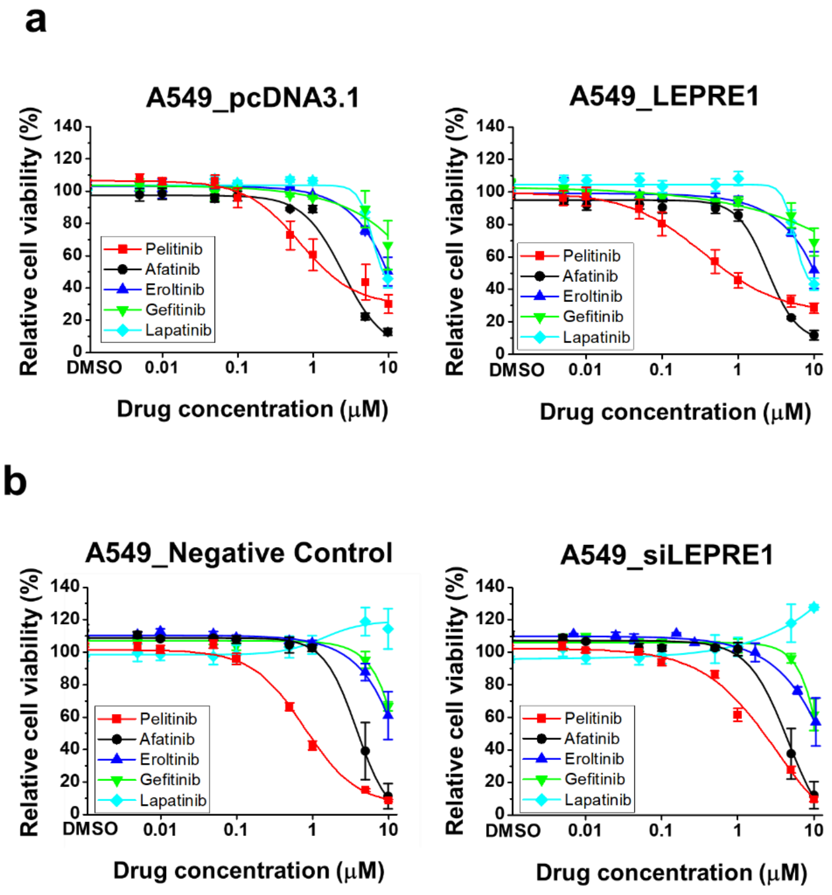

IC$_{50}$ values (μM)

EGFR-TKIs	pcDNA3.1	LEPRE1/pcDNA3.1	Negative Control	siLEPRE1
**Pelitinib**	1.141 ± 0.152	0.685 ± 0.088	0.784 ± 0.055	3.254 ± 0.960
**Afatinib**	2.540 ± 0.329	2.434 ± 2.711	3.853 ± 0.865	4.551 ± 1.436
**Erlotinib**	> 10	> 10	> 10	> 10
**Gefitinib**	> 10	> 10	> 10	> 10
**Lapatinib**	> 10	> 10	> 10	> 10

Supplementary Figure 3. Effect of EGFR-TKIs on A549_LEPRE1 and A549_siLEPRE1 cells

(a, b) A549 cells transfected with pcDNA3.1 and LEPRE1/pcDNA3.1 (**a**) or transfected or Negative control siRNA and siLEPRE1 (**b**) were incubated with EGFR-TKIs (afatinib, erlotinib, gefitinib, lapatinib, and pelitinib) for 72 h. Cell viability was determined using WST-1 proliferation assays. The IC$_{50}$ value of each condition is presented in the lower panel.

Supplementary Figure 4

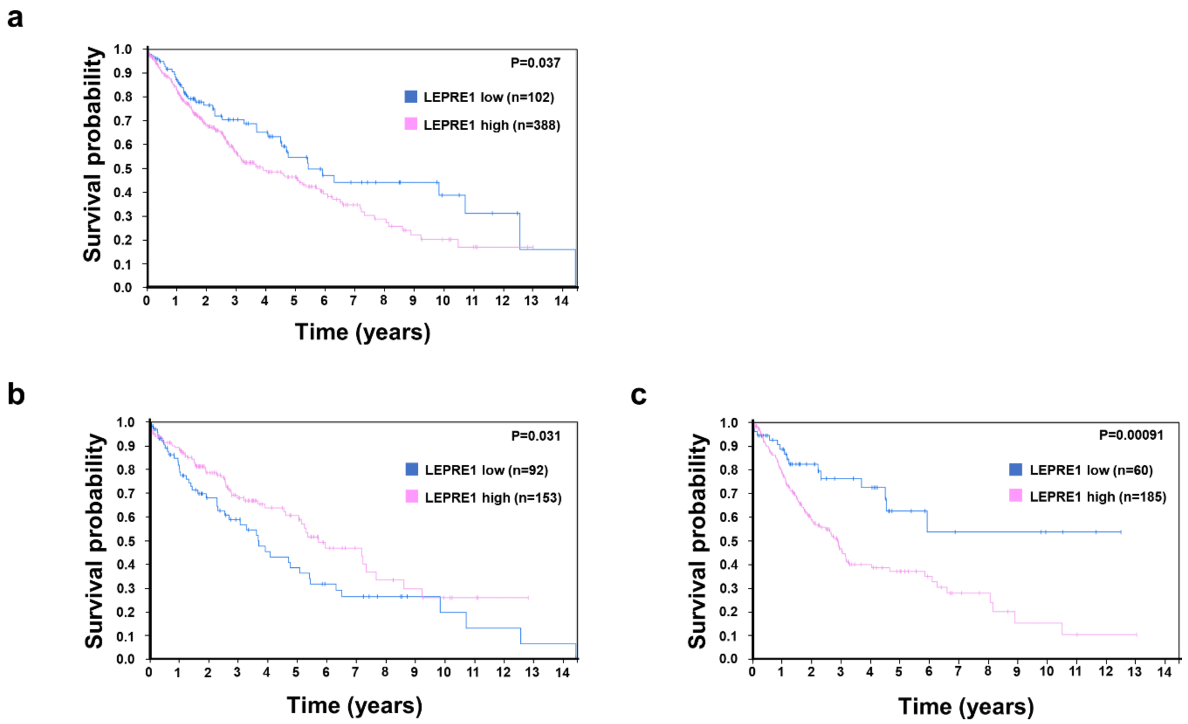

Supplementary Figure 4. Survival analysis of LEPRE1 in LUSC patients.

(**a**, **b**, **c**) Survival analysis of LEPRE1 in early and late clinical stage of lung squamous cell carcinoma (LUSC) patients from Human protein atlas. *Kaplan-Meier* curves for overall survival (**a**), early clinical stage (**b**)[stage i (n = 3), stage ia (n = 90), stage b (n = 149), stage ii (n = 3)], late clinical stage of cancer (**c**)[stage iia (n = 64), stage iib (n = 91), stage iii (n = 3), stage iiia (n = 62), stage iiib (n = 18)].

Full-length blots for cropped images

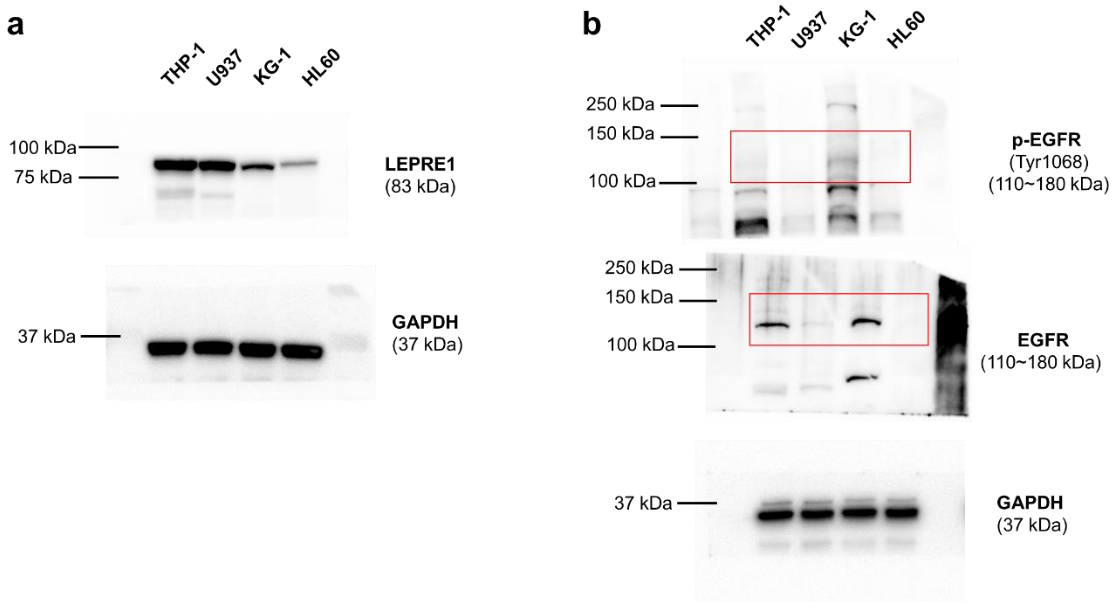

Supplementary Figure 5. Full-length blots of western blot figures shown in Figure 1. c, d.

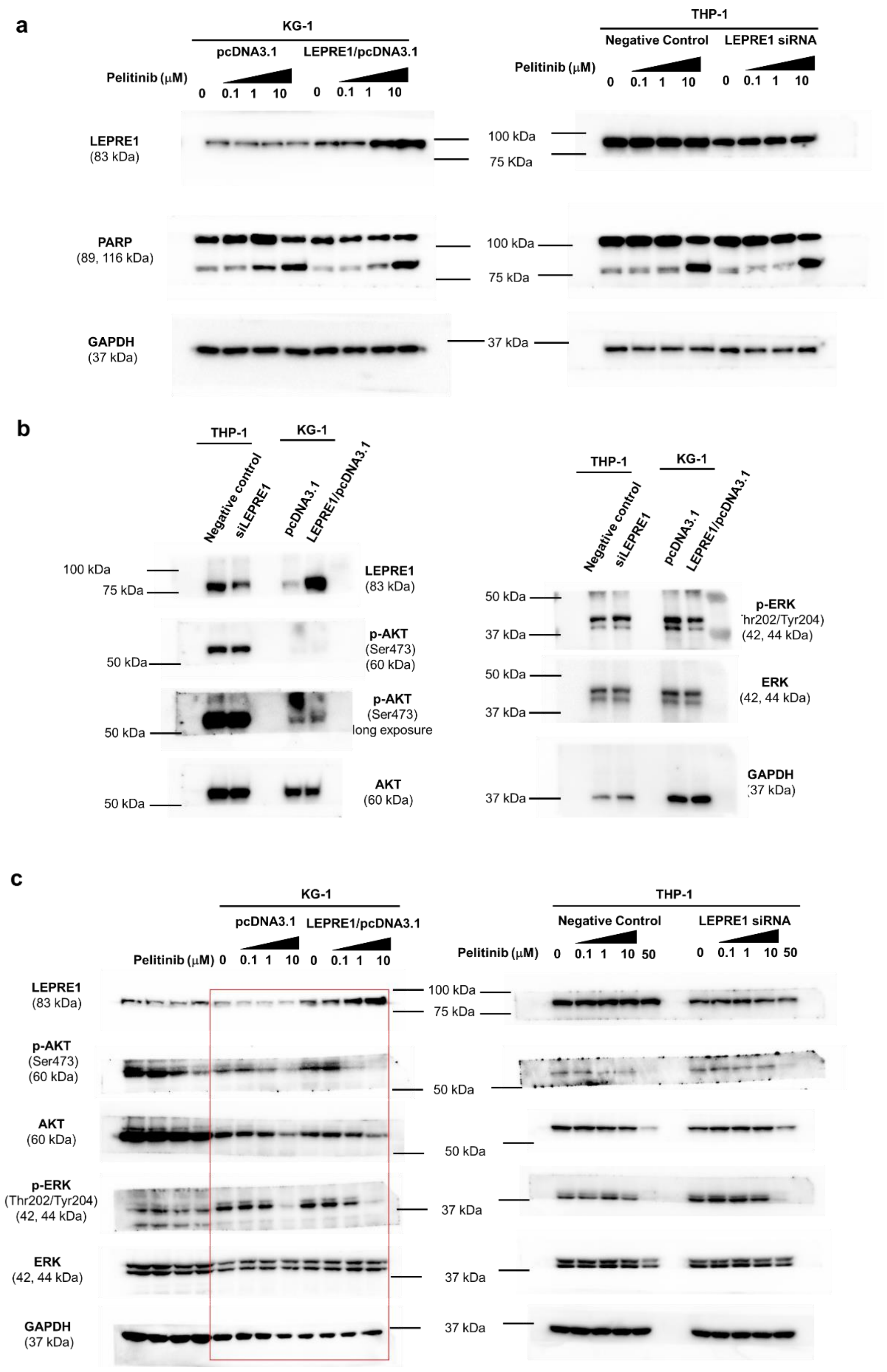

Supplementary Figure 6. Full-length blots of western blot figures shown in Figure 2. b, d, e, f, g

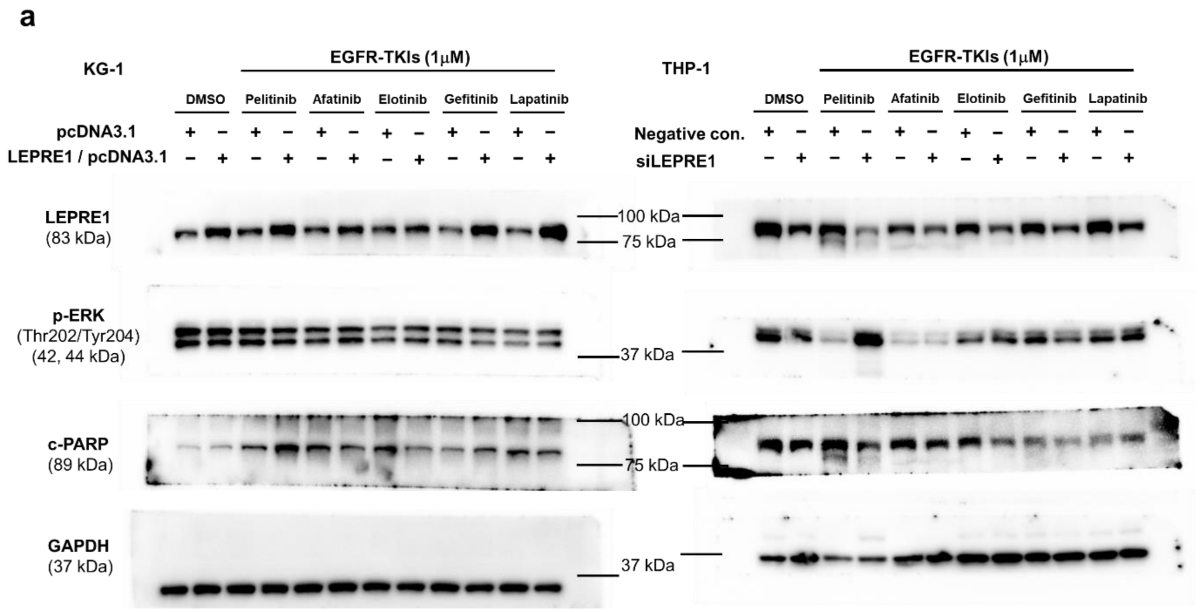

Supplementary Figure 7. Full-length blots of western blot figures shown in Figure 3. c, d.

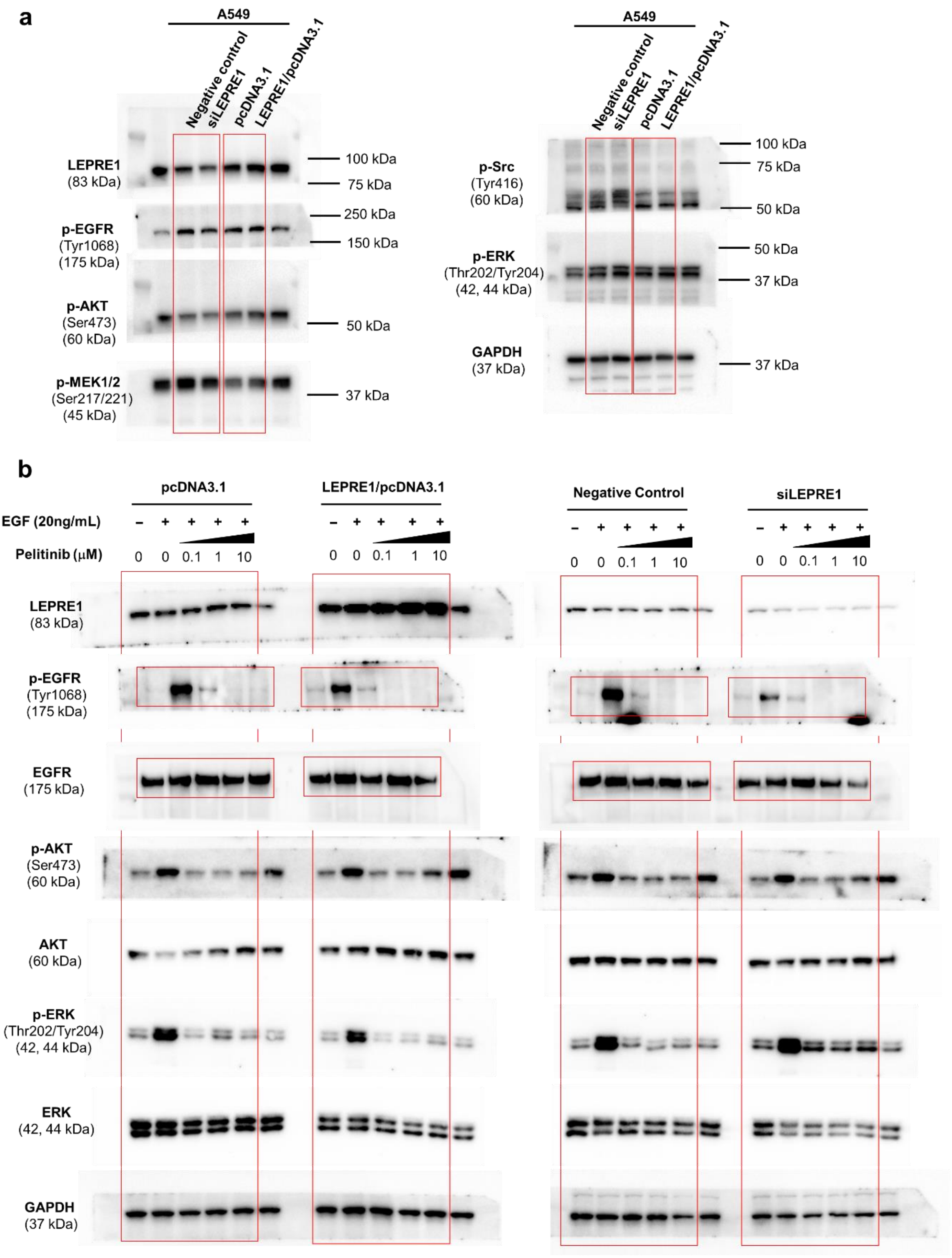

Supplementary Figure 8. Full-length blots of western blot figures shown in Figure 4. b, c, d.

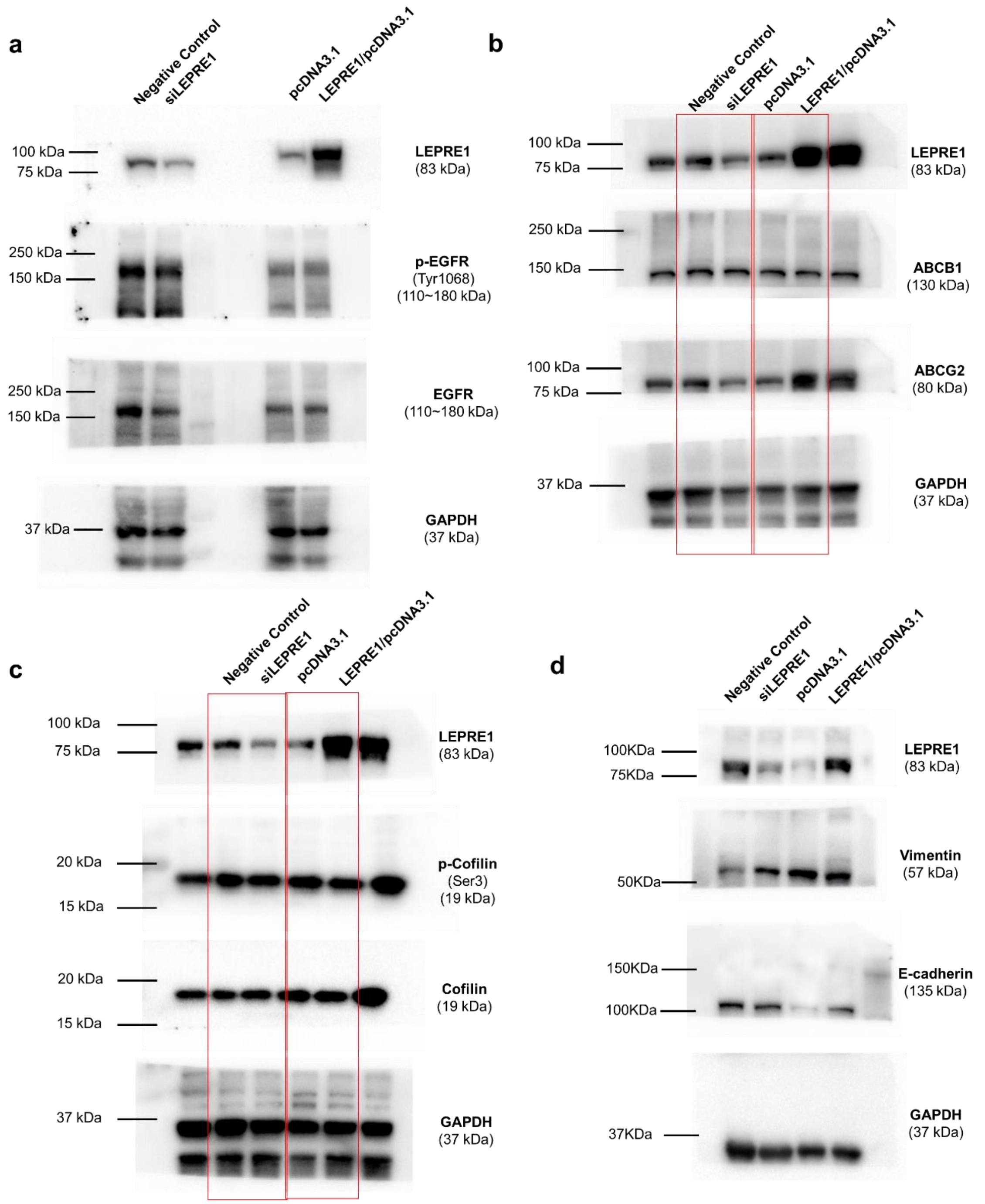

Supplementary Figure 9. Full-length blots of western blot figures shown in Figure 6. a, b, c, d.

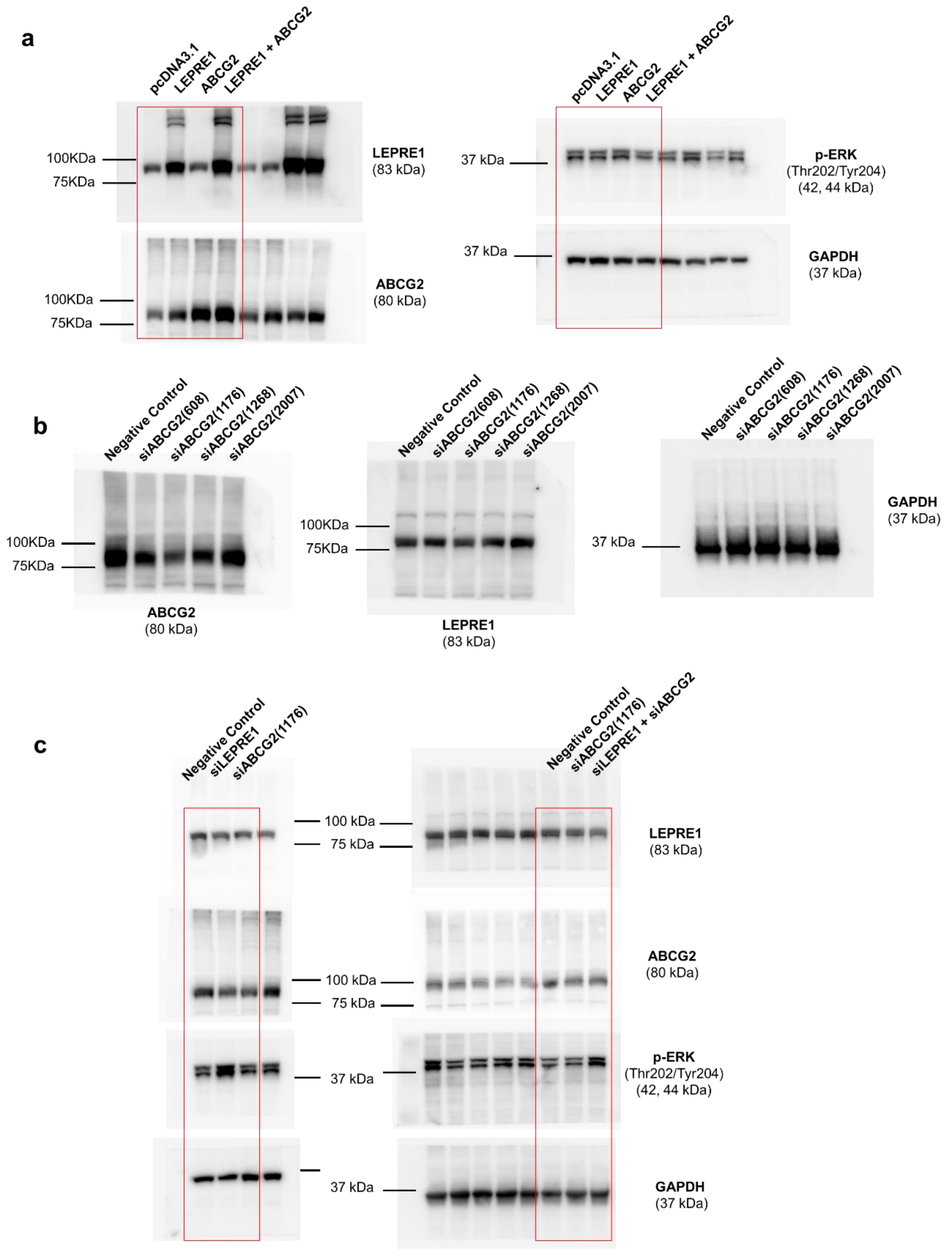

Supplementary Figure 10. Full-length blots of western blot figures shown in Figure 7. a, b, c.

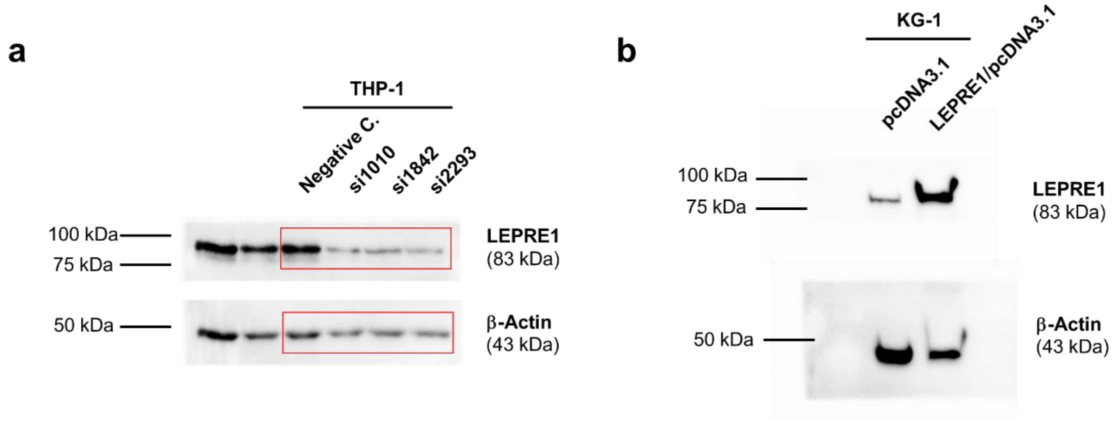

Supplementary Figure 10. Full-length blots of western blot figures shown in Supplemenatry Figure 2.
